# Validation, Replication, and Sensitivity Testing of Heckman-Type Selection Models to Adjust Estimates of HIV Prevalence

**DOI:** 10.1371/journal.pone.0112563

**Published:** 2014-11-17

**Authors:** Samuel J. Clark, Brian Houle

**Affiliations:** 1 Department of Sociology, University of Washington, Seattle, Washington, United States of America; 2 Institute of Behavioral Science, University of Colorado at Boulder, Boulder, Colorado, United States of America; 3 MRC/Wits Rural Public Health and Health Transitions Research Unit (Agincourt), School of Public Health, Faculty of Health Sciences, University of the Witwatersrand, Johannesburg, South Africa; 4 Australian Demographic and Social Research Institute, The Australian National University, Canberra, Australia; UNAIDS, Trinidad And Tobago

## Abstract

A recent study using Heckman-type selection models to adjust for non-response in the Zambia 2007 Demographic and Health Survey (DHS) found a large correction in HIV prevalence for males. We aim to validate this finding, replicate the adjustment approach in other DHSs, apply the adjustment approach in an external empirical context, and assess the robustness of the technique to different adjustment approaches. We used 6 DHSs, and an HIV prevalence study from rural South Africa to validate and replicate the adjustment approach. We also developed an alternative, systematic model of selection processes and applied it to all surveys. We decomposed corrections from both approaches into rate change and age-structure change components. We are able to reproduce the adjustment approach for the 2007 Zambia DHS and derive results comparable with the original findings. We are able to replicate applying the approach in several other DHSs. The approach also yields reasonable adjustments for a survey in rural South Africa. The technique is relatively robust to how the adjustment approach is specified. The Heckman selection model is a useful tool for assessing the possibility and extent of selection bias in HIV prevalence estimates from sample surveys.

## Introduction

HIV prevalence is commonly measured by collecting HIV biomarkers in household sample surveys. There are two selection processes that separate the population from individuals who agree to biomarker collection: (1) the ability to locate selected individuals to be interviewed, and (2) the consenting process for interviewed participants to collect HIV biomarkers. At both stages, the sample can vary systematically from the population, resulting in bias in population HIV prevalence estimates. For instance, those who already know their HIV status may be less likely to consent to HIV testing.

Heckman-type selection models [1] estimate and adjust for correlation between HIV status and the probability of participating in HIV testing. While these models are widely used in economics and other social sciences [2]–[5], they have been rarely applied in epidemiological studies – making their use controversial. Several recent papers have used Heckman-type selection models [1] to adjust for selective non-response in sample surveys [6]–[9], and Floyd and colleagues [10] have tried a variety of similar approaches to quantify the effects on non-response in HIV surveys.

Demographic and Health Surveys (DHSs), as population-based surveys, are widely used to estimate national HIV prevalence. However, these surveys may be subject to bias from selective nonresponse. A paper by Barnighausen et al. [6] applied Heckman-type selection models to the 2007 Zambia Demographic and Health Survey. They found a strong correction in male HIV prevalence that removed the gender disparity found in the original analyses [11]. This was largely due to an increase in the adjusted HIV prevalence for men from 12% to 21% [6].

Given the striking finding of a strong selection bias for men in the 2007 Zambia DHS, the accompanying recommendation to widely apply selection model methods to all DHSs, and the relative rarity of these methods being applied in epidemiological studies, in this study we aim to: (1) independently validate the reported finding for the 2007 Zambia DHS; (2) replicate the method to several DHSs; (3) apply the method to an external context with a population observed over time; and (4) explore the sensitivity of the method to alternate specification.

## Materials and Methods

### Ethics Statement

The sample survey in South Africa received ethical approvals from the University of the Witwatersrand Human Research Ethics Committee and the Mpumalanga Provincial Research and Ethics Committee. Ethics committee approval was not needed for the Demographic and Health Surveys work – all data were analyzed anonymously.

### Data

We apply the Heckman-type selection model and generate adjusted HIV prevalence for 5 DHSs: Lesotho 2004 – 05, Lesotho 2009 – 10, Swaziland 2006 – 07, Zambia 2007, and Zimbabwe 2005 – 06. These surveys were selected in order to apply the method under several different scenarios, including: relatively high non-response rate for the HIV test to allow for potentially greater influence of the selection model over the adjusted prevalence; in the same country close in time with different non-response rates, in order to compare measured and adjusted estimates; and a large gender disparity in measured HIV prevalence similar to the Zambia 2007 estimates.

We also use data collected from a health and demographic surveillance system (HDSS). The Agincourt HDSS is located in rural northeast South Africa. Since 1992 the study has conducted annual censuses of all households in 21 study villages. Vital events, migrations, and other information are collected at each census [12]. During 2010–11 we conducted a sample survey that collected data describing HIV and noncommunicable disease risk factors and biomarkers on a sex-age-stratified sample of 7,662 individuals from an eligible population of 34,413 individuals fifteen years old and older [13]. A research team visited sampled individuals up to 3 times for enrollment and informed consent. Cyclic labour migration is common in this population, especially for men – leading to differential nonresponse to the survey. These data represent an external context to apply Heckman-type selection models, where we have longitudinal data on individuals and a detailed understanding of the likely nonresponse processes at work.

For access to the Agincourt HDSS survey please contact Dr. F. Xavier Gómez-Olivé (Xavier@aingoucrt.co.za). For access to the DHSs please contact MEASURE DHS (http://www.measuredhs.com).

### Analyses

#### Replicating Bärnighausen et al.'s ‘2-stage’ approach for DHS surveys

For each DHS we apply the Heckman selection model and generate adjusted HIV prevalence following the approach used by Bärnighausen et al. [6] – which we call the ‘2-stage’ approach. We use a probit model for the outcome HIV status for individual 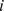


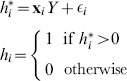
(1)where 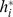 is an unobserved latent variable determining the likelihood of HIV infection, and depends on observed covariates 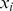 and random error 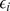.

We also use a probit model for selection 
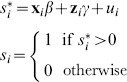
(2)where 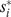 is an unobserved latent variable determining the likelihood of selection, and depends on observed covariates 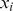, exclusion criteria 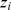, and random error 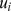. We observe 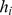 when 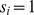. The estimated Heckman 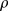 allows for correlation between the error terms in the outcome and selection equation 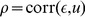
(3)


We consider two sources of non-response: (1) individuals who are unable to be contacted (the *contact* regression, which includes the entire eligible sample), and (2) individuals who are contacted but refuse HIV testing (the *consent* regression, which includes the eligible sample who were interviewed). We estimate these models separately for men and women, and include the same covariates used by Bärnighausen et al. [6]. We also specify the same exclusion criteria: for the contact regression we include household interviewer identity and if the household interview occurred on the first day of fieldwork in the cluster. For the consent regression, we include individual-interview interviewer identity. These are included since results are more robust if there are exclusion criteria that correlate with selection but are not correlated with the outcome.

For the sample survey data from the Agincourt HDSS we follow the same approach, considering non-response due to: (1) individuals who are unable to be contacted (the *contact* regression, which includes the eligible sample), and (2) individuals who are contacted but refuse HIV testing (the *consent* regression, which includes the eligible sample who were interviewed). The consent regression uses variables that would be available from an individual-level interview in a typical DHS-style cross-sectional survey. We specify the contact regression to include variables that would be available from a household-level interview in a typical DHS-stye cross-sectional survey. These are specified as in equations 1 and 2, where 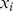 are based on observables from the survey and 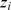 is the identity of the survey fieldworker.

The adjusted HIV prevalence is calculated using observed HIV status for the 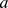 individuals 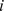 who were tested, the predicted probability of being HIV^+^ given not consenting for the 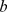 individuals 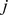 who were contacted but refused testing (from the consent regression) and the predicted probability of being HIV^+^ given not contacted for the 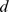 individuals 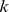 who were not contacted (from the contact regression). The Bärnighausen-method adjusted estimate of HIV prevalence 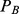 for the population 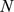 is 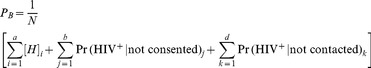
(4)


#### New ‘multi-stage’ adjustment method for Agincourt HDSS HIV survey

To make the following equations easier to read, we introduce new notation. For HIV^+^ we use 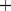, for those who were found 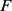, for those who were found and interviewed 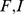, and for those who were found, interviewed, and tested 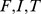. We use the negation operator 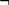 to indicate ‘the opposite of’ – i.e. 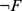 means ‘not found’.

As an alternative to the 2-stage approach we derive a new ‘multi-stage’ method. For the sample survey from the Agincourt HDSS we consider three reasons for nonresponse: (1) not being found, (2) (found but) not consenting to the interview, and (3) (found and interviewed but) not consenting to HIV testing. Based on a map of the outcome space that includes decision points 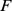 or 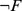, 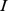 or 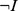, and 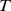 or 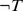, we define symmetric counterfactuals and model these using a combination of Heckman selection models and imputation.

We know the HIV status 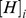 of those who were found, interviewed and tested 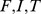. For those who where found and interviewed but did not consent for testing 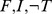 we use the probit models specified in equations 1 and 2 with the identity of the interviewer as the exclusion criteria 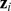. The predicted probability of being HIV^+^ in the 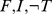 group is 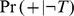.

We next consider those who were found but did not agree to be interviewed, the 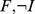 group. The counterfactual for this group divides them into tested 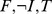 and not tested 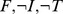, and we use a Heckman selection model to predict the probability of being tested given that a respondent refused to be interviewed. We model the outcome ‘being tested’ for individual 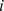 with the probit model 
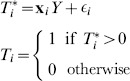
(5)where 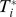 is an unobserved latent variable determining the likelihood of being tested as a function of observed covariates 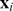 and a random error 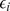. We model selection into ‘being interviewed’ with the probit model 
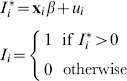
(6)where 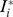 is an unobserved latent variable determining the likelihood of being interviewed as a function of observed covariates 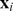 and a random error 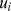. We observe 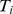 when 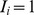. The estimated Heckman 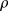 allows for correlation between the error terms in the outcome and selection equation as specified in equation 3. This biprobit Heckman selection model is estimated on everyone who was found 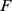. The predicted probability of being tested in the 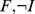 subgroup is 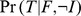. We use this probability to divide the 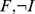 into 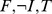 and 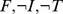 groups. To predict the HIV status of those in the tested and not tested subgroups, we assume that they are HIV^+^ in proportions equal to those in the 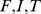 group who actually had a positive test and those who are predicted to be positive in the 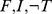 (just above). We impute these values using probabilities predicted from equations 1 and 2 for the counterfactual 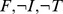 group and observed HIV status 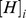 in the observed 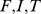 group for the counterfactual 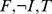 group.

Finally we consider those who were not found for an interview at all, the 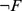 group. The full counterfactual for this group divides them into interviewed 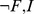 and not interviewed 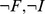, and further into tested 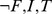 and not tested 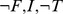 among those who are interviewed and tested 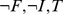 and not tested 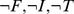 among those who are not interviewed. We use a Heckman selection model to predict the probability of being interviewed given that a respondent was not found. We model the outcome ‘being interviewed’ for individual 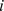 with the probit model 
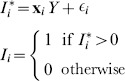
(7)where 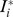 is an unobserved latent variable determining the likelihood of being interviewed as a function of observed covariates 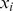 and a random error 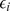. We model selection into ‘being found’ with the probit model 
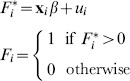
(8)where 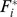 is an unobserved latent variable determining the likelihood of being found as a function of observed covariates 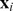 and a random error 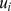. We observe 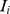 when 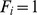. The estimated Heckman 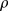 allows for correlation between the error terms in the outcome and selection equation as specified in equation 3. This biprobit Heckman selection model is estimated on the entire eligible sample. To predict the probabilities of being HIV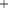 in the four tested subgroups in this counterfactual (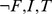, 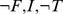, 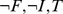 and 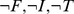), we follow exactly the same logic as described just above for the 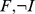 group, with the additional level of found/not found. The final multi-stage adjusted population HIV prevalence 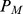 is 
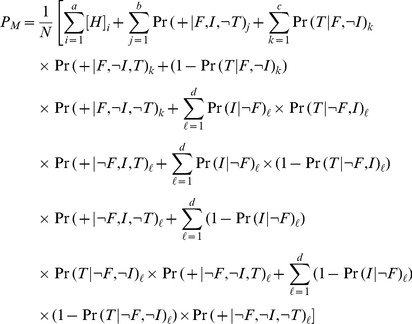
(9)where 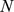 is the total number of individuals in the population, 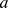 is the number in the 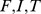 group; 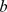 the number in the 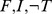 group; 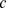 the number in the 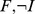 group and 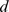 the number in the 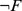 group.

#### Application of the multi-stage correction method to DHS surveys

In order to compare the two adjustment methods using DHS data, we also apply the multi-stage adjustment method to the five DHS surveys for which we estimate adjusted HIV prevalences using the Bärnighausen 2-stage method. Because the DHS surveys do not contain information that allows us to model the outcome ‘being tested’, the multi-stage method for DHS surveys requires only two models:

1. Predicting the probability of being HIV^+^ among those interviewed. We use equations 1 and 2, using interviewer identity as the exclusion criteria.

2. Predicting the probability of being interviewed among those contacted. We use equations 7 and 8 with the exclusion criteria being the number of household visits.

We calculate adjusted population HIV prevalence in a manner analogous to equation 10. The final multi-stage adjusted population HIV prevalence for DHS surveys is 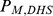 is 
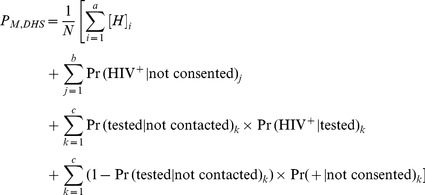
(10)where 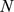 is the total number of individuals in the population, 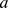 is the number of individuals who consented to HIV testing indexed by 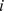; 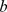 the number of individuals contacted indexed by 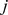; 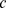 the number of individuals not contacted indexed by 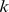.

#### Decomposing differences in crude prevalence rates

Like all ‘crude’ rates, the overall population prevalence of HIV is a weighted average across dimensions along which HIV prevalence varies; sex and age being two of the important ones. The differences between crude rates – the adjustments we are calculating with these methods – are the result of changes in the prevalence profiles across these subgroups *and* changes in the composition of the population across the subgroups. In our case, the sex-age profile of prevalence may change to bring about the difference, *or* the sex-age composition of the population may change to provide different weights for the same sex-age profile of prevalence. To unravel how much of each type of change is contributing to the overall difference, we can decompose the adjustments to overall population-average HIV prevalence rates into components resulting from changes in the sex-age prevalence rates and the sex-age composition of the population. To do this we use standard methods described by Preston and coauthors [14].

## Results

### Application to the DHS

**Figure 1:**
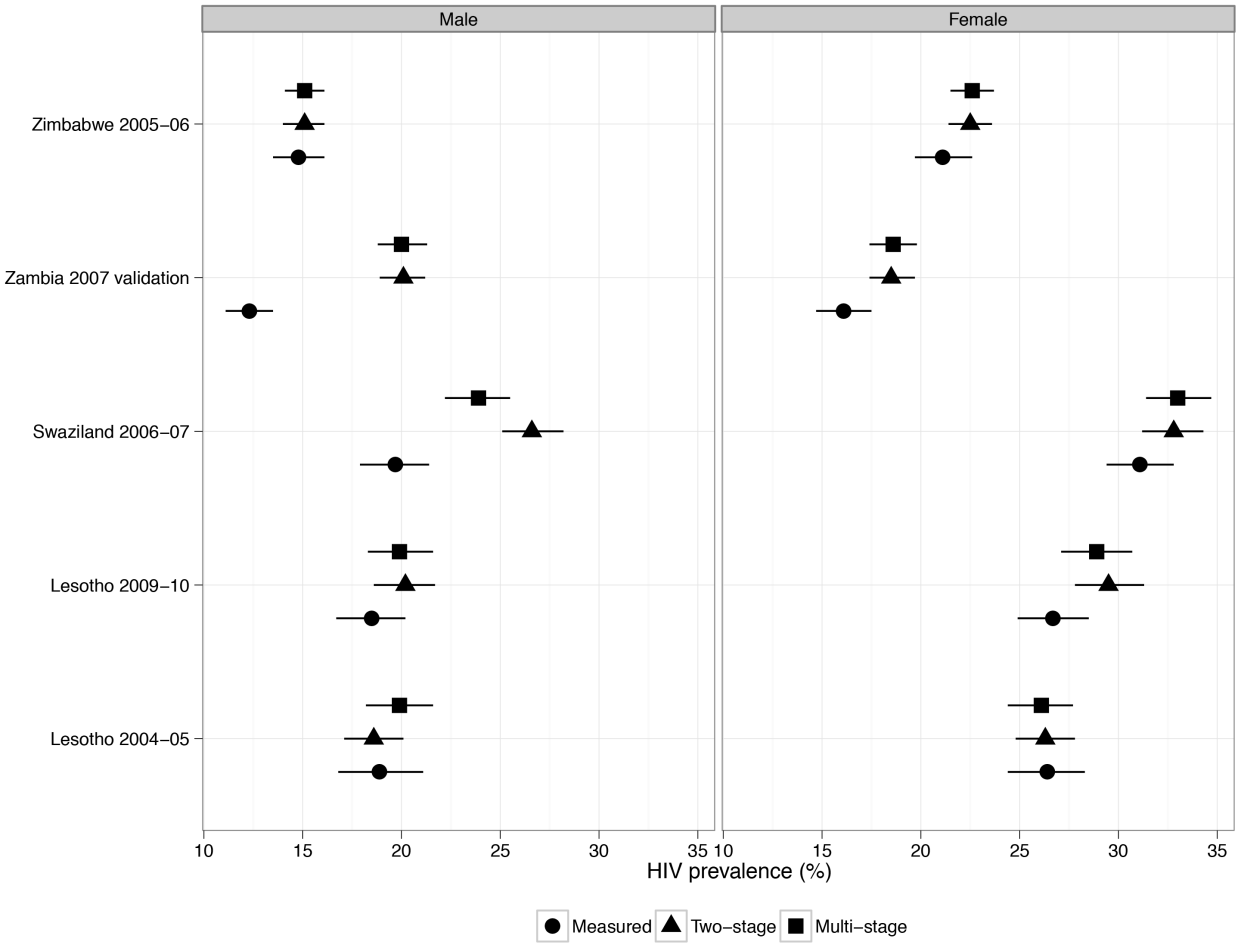
Measured and adjusted HIV prevalence for 5 Demographic and Health Surveys, by sex and 2-stage and multi-stage approaches. Values use survey weights and take into account survey design. Weights are normalized by dividing by 1,000,000. We apply weights specific to the HIV sample to generate ‘Measured’ prevalence for comparison purposes. We apply household weights to each individual in calculating the ‘Multi-stage’ and ‘2-stage’ HIV prevalence.

Regression outputs are available in supporting information Tables S1. Figure 1 and Table 1 show the adjusted HIV prevalence results using the 2-stage approach of Bänighausen et al. for the DHS. Our 2007 Zambia results closely align to those found in the original paper [6]: we find a corrected HIV prevalence for males of 20.1% compared to their 21% and a corrected prevalence for females of 18.5% compared to their 18%. Both results find a small correction in HIV prevalence for women and a large correction in male HIV prevalence. The remaining difference of 0.09 percentage points may be due to differences in the coding of analytic variables.

**Table 1:** Measured and adjusted HIV prevalence for 5 Demographic and Health Surveys.

		Measured		Two-stage		Multi-stage	
Survey	Sex	%	95% CI	%	95% CI	%	95% CI
Zimbabwe 2005–06	Male	14.8	(13.5,16.1)	15.1	(14.0,16.1)	15.1	(14.1,16.1)
Zambia 2007 validation	Male	12.3	(11.1,13.5)	20.1	(18.9,21.2)	20.0	(18.8,21.3)
Swaziland 2006–07	Male	19.7	(17.9,21.4)	26.6	(25.1,28.2)	23.9	(22.2,25.5)
Lesotho 2009–10	Male	18.5	(16.7,20.2)	20.2	(18.6,21.7)	19.9	(18.3,21.6)
Lesotho 2004–05	Male	18.9	(16.8,21.1)	18.6	(17.1,20.1)	19.9	(18.2,21.6)
Zimbabwe 2005–06	Female	21.1	(19.7,22.6)	22.5	(21.4,23.6)	22.6	(21.5,23.7)
Zambia 2007 validation	Female	16.1	(14.7,17.5)	18.5	(17.4,19.7)	18.6	(17.4,19.8)
Swaziland 2006–07	Female	31.1	(29.4,32.8)	32.8	(31.2,34.3)	33.0	(31.4,34.7)
Lesotho 2009–10	Female	26.7	(24.9,28.5)	29.5	(27.8,31.3)	28.9	(27.1,30.7)
Lesotho 2004–05	Female	26.4	(24.4,28.3)	26.3	(24.8,27.8)	26.1	(24.4,27.7)

Values use survey weights and take into account survey design. Weights are normalized by dividing by 1,000,000. We apply weights specific to the HIV sample to generate ‘Measured’ prevalence for comparison purposes. We apply household weights to each individual in calculating the ‘Multi-stage’ and ‘2-stage’ HIV prevalence.

For Lesotho in Figure 1 and Table 1 we have two time points with different response rates. In the later survey the response rate increased (82% of women and 71% of men in 2004 [15]; 94% of women and 88% of men in 2009 [16]) but measured HIV prevalence remained relatively stable (26% of women and 19% of men in 2004 [15]; 27% of women and 19% of men in 2009 [16]). In this case measured HIV prevalence does not respond to changes in response rate, suggesting that nonresponse bias was small in the earlier survey with a higher nonresponse rate. The adjusted prevalence correction is correspondingly small for both men and women in the earlier survey. While the adjusted prevalence correction is larger in the more recent survey, the low nonresponse rates made model convergence difficult.

For Zimbabwe in Figure 1 and Table 1 there was a moderate degree of nonresponse (77% of men and 84% of eligible women were covered) [17]. The adjusted prevalence correction is quite minor. The results from Swaziland show a large male HIV prevalence correction that reduces the gender gap from 11 percentage points higher for women to 6 percentage points higher for women.

Figure 1 and Table 1 also show the results comparing the 2-stage and multi-stage approaches. The results are very similar for most of the surveys. For males in the Swaziland 2006 – 07 DHS the multi-stage estimate is approximately 2.7 percentage points lower than the 2-stage estimate. For males in the Lesotho 2004 – 05 DHS the multi-stage estimate is approximately 1 percentage point higher than the 2-stage estimate. These two surveys have a relatively larger amount of nonresponse due to individuals who were not contacted, which allows the different adjustment techniques to vary slightly.

### Application to the Agincourt HDSS

Regression outputs are available in supporting information Tables S2. Measured HIV prevalence was 19.4%; 23.9% for females and 10.6% for males. The 2-stage approach adjusted HIV prevalence was 22.1%; 25.4% for females and 16.9% for males). The multi-stage approach adjusted HIV prevalence was 23.1%; 26.9% for females and 17.1% for males.

**Figure 2:**
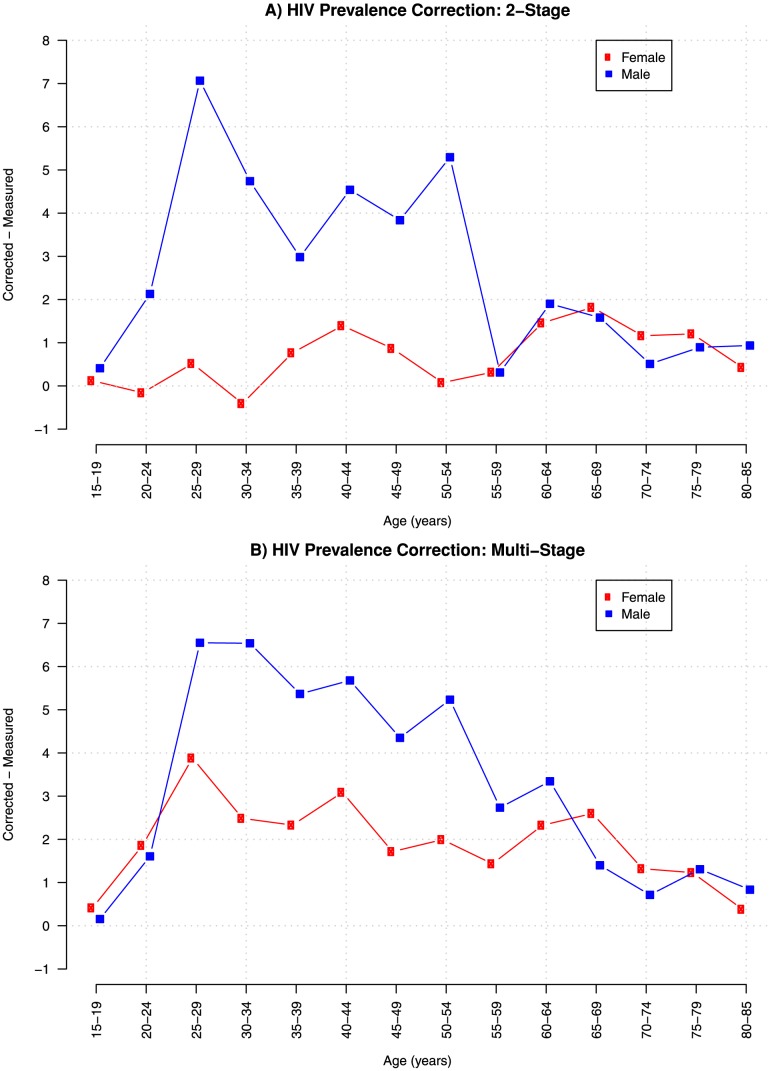
Corrections to sex-age specific Agincourt HDSS HIV prevalence, 2010 – 2011. A) Corrections using 2-stage approach; B) Corrections using multi-stage approach.

The corrections shown in Figure 2 are the differences between measured HIV sex-age-specific prevalence and the adjustments from the multi- and 2-stage approaches. The 2-stage approach increases overall HIV prevalence by 2.7 percentage points; 1.5 percentage points for females and 6.3 percentage points for males. The multi-stage approach increases overall HIV prevalence by 3.6 percentage points; 3 percentage points for females and 6.4 percentage points for males.

**Figure 3:**
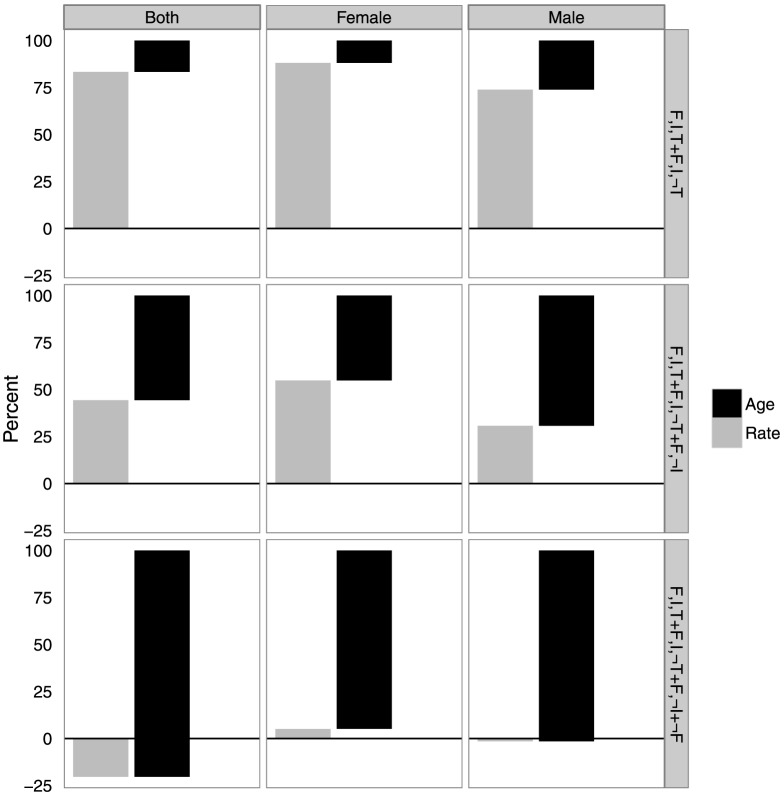
Decomposition of sub-group crude rates into rate and age composition differences using the multi-stage approach, Agincourt HDSS 2010 – 2011. Rate and age differences sum to 100% for each sex subgroup. For those who were found 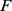, for those who were found and interviewed 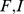, and for those who were found, interviewed, and tested 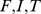. We use the negation operator 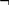 to indicate ‘the opposite of’ – i.e. 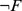 means ‘not found’.

Figure 3 displays the decomposed crude rates into rate and age composition differences by subsequently adding each group using the multi-stage approach. The rate and age differences sum to 100% for each sex subgroup. When adding the not-tested to the tested subgroup (which increases prevalence by 1.9 percentage points) the sex-age prevalence is the larger component of the difference. Adding the not-interviewed group (which increases prevalence by 0.7 percentage points) the two components contribute similarly to the difference. Finally, adding the not-found group (which increases prevalence by 1.1 percentage points) the sex-age composition of the population represents almost all of the difference.

**Figure 4:**
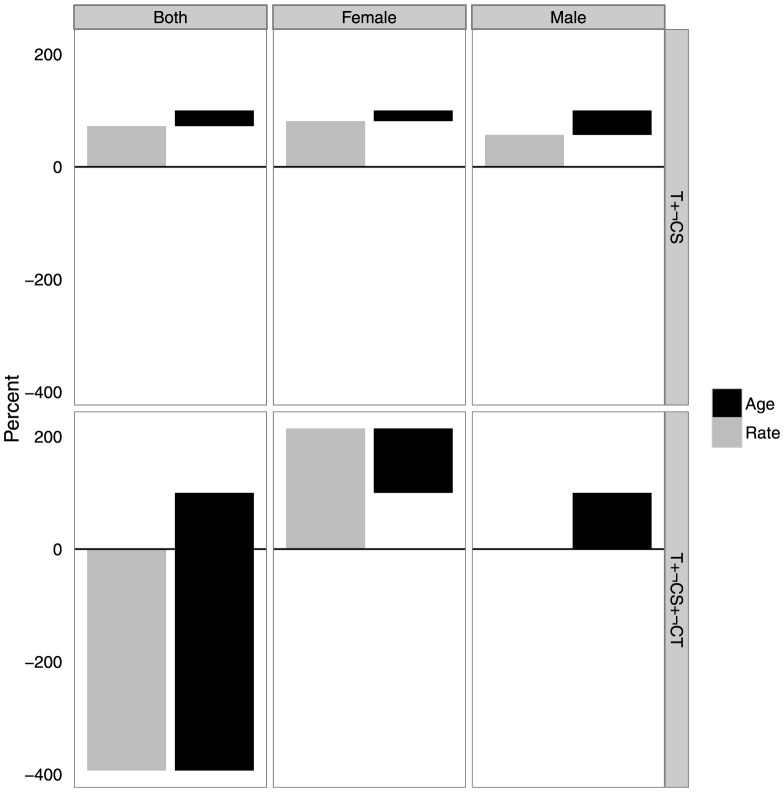
Decomposition of sub-group crude rates into rate and age composition differences using the 2-stage approach, Agincourt HDSS 2010 – 2011. Rate and age differences sum to 100% for each sex subgroup. For those who were contacted we use Ct, for those who consented we use CS, and for those who were tested T. We use the negation operator 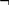 to indicate ‘the opposite of’ – i.e. 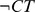 means ‘not contacted’.

Figure 4 displays the decomposed crude rates into rate and age composition differences by subsequently adding each group using the 2-stage approach. Adding the not-consenting to the test subgroup (which increases prevalence by 2.4 percentage points) the sex-age prevalence and sex-age composition both provide positive contributions to the difference. For females, adding the not-contacted group the sex-age prevalence contributes twice the magnitude of overall change in population prevalence. Age composition changes operate in the opposite direction to decrease the change in population prevalence. For males, age-composition contributes nearly all of the difference in population prevalence.

## Discussion

We were able to validate the original findings for the Zambia 2007 DHS [6], and replicate the method across several other DHSs. We also found the DHS adjusted results to be relatively robust to different methods to calculate the adjusted prevalence.

The adjustments for the Agincourt HDSS indicated a larger correction for males, which integrates with our understanding of male nonresponse due to cyclical migration. The adjustment was also relatively robust to each method of calculating adjusted prevalence, with slightly higher corrections for females using the multi-stage approach. For both the multi-stage and 2-stage approaches most of the correction for females was due to the model of self-selection into testing. Most of this correction was due to differences in age-specific rates rather than differences in age composition. For males the non-testing and not-found groups contributed about equally. For male non-testers most of the contribution was from age-specific prevalence rates. For not-found males, the changes in prevalence were driven almost entirely by differences in age structures of the found and not-found populations.

Our study has limitations. First, we ignored uncertainty from the underlying 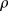 parameter when calculating adjusted HIV prevalence (i.e., when calculating 95% CIs we estimate sampling uncertainty conditional on the estimated regression parameters). However, our main goal was to conduct an independent validation of the original findings [6]. Future work is needed to incorporate uncertainty from the model-based adjustment – Hogan et al. employed a parametric simulation approach [9]. Second, the multi-stage approach assumes that the imputed conditional probabilities are similar in the observed and unobserved situations. The 2-stage approach also assumes that those who do not consent would follow a similar nonresponse pattern relative to those who were not contacted. While each approach makes untestable assumptions, they yield similar results.

Our results suggest that Heckman-type selection models are useful for epidemiological studies to assess the importance of selection bias in the population parameter of interest and how sensitive the parameter is to selective nonresponse. Our independent validation produced remarkably similar findings to the original paper [6], and the results are relatively robust to different approaches to adjusting HIV prevalence. Future work is needed to determine how to calculate adjusted HIV prevalence in light of all available evidence (both measured and modeled). Ultimately the choice of selection model will be problem-specific and dependent on the researchers' and modeling assumptions, as well as the data available [18].

Based on these results we recommend that all surveys, including DHS, that include HIV testing calculate and present both unadjusted and Heckman biprobit-adjusted estimates of HIV prevalence. Heckman biprobit-based adjustments can be made using either the two-stage or multi-stage approach, but we prefer the multi-stage approach because it faithfully replicates the selection steps involved in identifying the final sample of people who agree to testing, and moreover, the multi-stage approach can easily be modified to accommodate more or less complex selection hierarchies. The magnitude of the difference between the unadjusted and adjusted estimates is a rough indicator of how consequential selective nonresponse may be. Large differences suggest important effects of selective nonresponse and suggest that both estimates should be interpreted with caution. To improve the reliability of the Heckman biprobit adjustment methods, surveys should record detailed information describing the field workers (e.g. age, sex, experience, ethnicity, etc.) and operational logistics (e.g. which field teams operate in which areas and when, etc.). Information like this can be used to construct good selection variables, and additionally in a completely different sense, to investigate and possibly control for interviewer effects in general.

## Supporting Information

Tables S1 **Consent and contact regressions for 5 Demographic and Health Surveys.**
(PDF)

Tables S2 **Regressions for the Agincourt health and demographic surveillance system.**
(PDF)
