# Supplementary material for: Validation, Replication, and Sensitivity Testing of Heckman-Type Selection Models to Adjust Estimates of HIV Prevalence
Source: PLoS One. 2014 Nov 17;9(11):e112563. doi: 10.1371/journal.pone.0112563 (PMC4234405; doi:10.1371/journal.pone.0112563)
Supplement: Tables S1 — Consent and contact regressions for 5 Demographic and Health Surveys. (PDF) [file pone.0112563.s001.pdf]

Margins from DHS Consent and Contact Regressions, by Gender

* The original code is available **here** from: David Canning; Till Brnighausen, 2011, "Replication data for: Heckman selection model to correct HIV prevalence estimates for survey non participation", http://hdl.handle.net/1902.1/17657 V3 [Version]. Code for the 'multi-stage' approach are available from the authors upon request.

Table 1. Zambia 2007 Contact Regression Marginals, Female

	dy/dx	95% CI
Age category		
20 – 24	0.096	0.050, 0.142
25 – 29	0.174	0.120, 0.228
30 – 34	0.216	0.152, 0.279
35 – 39	0.224	0.158, 0.291
40 – 44	0.175	0.118, 0.231
45 – 49	0.127	0.076, 0.179
Wealth quintile		
2nd	0.023	-0.017, 0.062
3rd	0.049	0.009, 0.089
4th	0.076	0.029, 0.123
5th	0.045	-0.006, 0.096
Mean education	0.004	0.000, 0.008
Location		
Small city	0.057	-0.032, 0.147
Town	0.005	-0.083, 0.093
Countryside	−0.070	-0.160, 0.019
Region		
Copperbelt	−0.084	-0.139, -0.028
Eastern	−0.073	-0.148, 0.002
Luapula	−0.086	-0.160, -0.012
Lusaka	−0.037	-0.126, 0.052
Northern	−0.122	-0.196, -0.049
Northwestern	−0.103	-0.170, -0.036
Southern	−0.056	-0.115, 0.002
Western	−0.009	-0.066, 0.049
Age category		
20 – 24	−0.012	-0.040, 0.016
25 – 29	0.022	-0.007, 0.051
30 – 34	0.033	0.003, 0.063
35 – 39	0.019	-0.019, 0.058
40 – 44	0.036	-0.005, 0.077
45 – 49	0.046	0.004, 0.088
Wealth quintile		

continued on next page

continued from previous page

	dy/dx	95% CI
2nd	0.001	-0.037, 0.040
3rd	−0.002	-0.044, 0.040
4th	0.022	-0.027, 0.072
5th	0.013	-0.046, 0.073
Mean education	0.006	0.002, 0.010
Location		
Small city	0.215	0.086, 0.343
Town	0.176	0.069, 0.283
Countryside	0.183	0.077, 0.289
Region		
Copperbelt	−0.155	-0.291, -0.018
Eastern	0.156	0.005, 0.307
Luapula	−0.123	-0.311, 0.065
Lusaka	0.048	-0.128, 0.224
Northern	−0.018	-0.163, 0.127
Northwestern	−0.156	-0.476, 0.164
Southern	0.034	-0.156, 0.223
Western	0.111	-0.114, 0.336
Interviewer 2	−0.047	-0.157, 0.063
Interviewer 3	−0.049	-0.152, 0.054
Interviewer 4	−0.134	-0.245, -0.022
Interviewer 5	−0.042	-0.160, 0.075
Interviewer 6	0.085	-0.038, 0.208
Interviewer 7	−0.016	-0.144, 0.112
Interviewer 8	0.061	-0.115, 0.237
Interviewer 9	0.133	0.011, 0.255
Interviewer 10	0.111	-0.036, 0.259
Interviewer 11	0.164	0.027, 0.301
Interviewer 12	−0.013	-0.134, 0.109
Interviewer 13	0.057	-0.084, 0.198
Interviewer 14	−0.130	-0.256, -0.004
Interviewer 15	−0.120	-0.246, 0.005
Interviewer 16	−0.037	-0.157, 0.083
Interviewer 17	−0.044	-0.163, 0.075
Interviewer 18	−0.073	-0.225, 0.079
o.Interviewer 19	0.000	0.000, 0.000
Interviewer 20	0.242	0.076, 0.409
Interviewer 21	0.113	-0.056, 0.282
Interviewer 22	0.114	-0.078, 0.307
Interviewer 23	0.206	0.047, 0.365
Interviewer 24	0.248	0.042, 0.455
Interviewer 25	0.271	0.076, 0.466
Interviewer 26	0.119	-0.064, 0.302
Interviewer 27	0.058	-0.087, 0.202
Interviewer 28	0.104	-0.046, 0.254

continued on next page

continued from previous page

	dy/dx	95% CI
Interviewer 29	−0.032	-0.172, 0.107
Interviewer 30	0.141	-0.014, 0.295
Interviewer 31	0.101	-0.077, 0.279
Interviewer 32	0.057	-0.076, 0.190
Interviewer 33	−0.001	-0.141, 0.139
Interviewer 34	−0.275	-0.424, -0.125
Interviewer 35	0.187	0.050, 0.323
Interviewer 36	0.239	0.059, 0.418
Interviewer 37	0.082	-0.082, 0.247
Interviewer 38	0.266	-0.034, 0.566
Interviewer 39	0.253	0.094, 0.413
Interviewer 40	−0.059	-0.187, 0.069
Interviewer 41	−0.003	-0.139, 0.132
Interviewer 42	−0.059	-0.217, 0.098
Interviewer 43	−0.078	-0.252, 0.097
Interviewer 44	−0.069	-0.204, 0.066
Interviewer 45	0.145	-0.147, 0.438
Interviewer 46	0.173	-0.137, 0.483
Interviewer 47	0.231	-0.072, 0.534
Interviewer 48	0.029	-0.305, 0.363
Interviewer 49	0.007	-0.287, 0.302
Interviewer 50	0.049	-0.246, 0.344
Interviewer 51	0.019	-0.172, 0.211
Interviewer 52	−0.052	-0.223, 0.118
Interviewer 53	−0.073	-0.243, 0.098
Interviewer 54	−0.042	-0.263, 0.179
Interviewer 55	−0.061	-0.265, 0.143
Interviewer 56	−0.099	-0.302, 0.104
First day	0.055	0.029, 0.080

N=7389, Censored cases=1683

ρ =-0.045, χ^2 =0.016, p=0.898

Table 2. Zambia 2007 Consent Regression Marginals, Female

	dy/dx	95% CI
Age category		
20 – 24	0.069	0.027, 0.112
25 – 29	0.163	0.113, 0.212
30 – 34	0.208	0.157, 0.260
35 – 39	0.216	0.163, 0.270
40 – 44	0.157	0.104, 0.209
45 – 49	0.099	0.045, 0.152
Wealth quintile		
2nd	0.008	-0.034, 0.050
3rd	0.033	-0.010, 0.077
4th	0.067	0.016, 0.118
5th	0.037	-0.020, 0.095
Mean education	0.003	-0.002, 0.007
Location		
Small city	0.044	-0.053, 0.140
Town	0.006	-0.087, 0.099
Countryside	−0.061	-0.148, 0.026
Region		
Copperbelt	−0.083	-0.146, -0.020
Eastern	−0.099	-0.173, -0.025
Luapula	−0.089	-0.162, -0.016
Lusaka	−0.060	-0.153, 0.033
Northern	−0.146	-0.219, -0.074
Northwestern	−0.108	-0.285, 0.069
Southern	−0.098	-0.166, -0.031
Western	−0.126	-0.292, 0.039
Religion		
Protestant	0.016	-0.013, 0.045
Muslim	−0.138	-0.416, 0.139
Ethnicity		
Lunda (L)	−0.035	-0.136, 0.066
Lala	0.023	-0.063, 0.110
Ushi	−0.071	-0.172, 0.031
Lamba	0.057	-0.027, 0.140
Tonga	−0.050	-0.105, 0.004
Luvale	0.041	-0.041, 0.124
Lunda (NW)	−0.006	-0.111, 0.098
Mbunda	−0.047	-0.136, 0.042
Kaonde	−0.016	-0.095, 0.063
Lozi	0.035	-0.024, 0.094
Chewa	0.041	-0.024, 0.106
Nsenga	0.086	0.030, 0.143

continued on next page

continued from previous page

	dy/dx	95% CI
Ngoni	0.042	-0.025, 0.108
Mambwe	0.038	-0.043, 0.119
Namwanga	−0.054	-0.130, 0.022
Tumbuka	−0.034	-0.103, 0.035
Other	0.017	-0.027, 0.062
Knows someone who died of AIDS	−0.018	-0.042, 0.006
Would care for relative with AIDS	0.054	-0.010, 0.118
Age at first sex		
≤15	0.176	0.118, 0.233
>15	0.165	0.106, 0.224
Married	−0.114	-0.164, -0.064
Drinks alcohol	0.062	0.026, 0.097
Ever tested for HIV	0.029	0.007, 0.052
Number of partners in last 12 months		
One	0.041	-0.021, 0.103
Multiple	0.074	-0.037, 0.185
STD in last 12 months	0.109	0.067, 0.151
Smokes tobacco	−0.099	-0.223, 0.025
High risk sex in last 12 months	−0.042	-0.099, 0.015
Condom use at last sex	0.066	0.030, 0.102
Interview language		
Bemba	0.029	-0.033, 0.091
Lozi	0.102	-0.052, 0.255
Nyanja	0.002	-0.060, 0.064
Tonga	0.078	0.009, 0.147
Other	0.013	-0.133, 0.159
Age category		
20 – 24	−0.012	-0.046, 0.022
25 – 29	0.005	-0.031, 0.041
30 – 34	0.011	-0.027, 0.048
35 – 39	0.012	-0.031, 0.055
40 – 44	0.007	-0.037, 0.051
45 – 49	0.029	-0.018, 0.076
Wealth quintile		
2nd	−0.001	-0.037, 0.034
3rd	−0.005	-0.042, 0.033
4th	0.031	-0.015, 0.078
5th	0.049	-0.005, 0.103
Mean education	0.006	0.002, 0.010
Location		
Small city	0.185	0.060, 0.310
Town	0.153	0.046, 0.260
Countryside	0.162	0.056, 0.268
Region		
Copperbelt	0.069	-0.274, 0.411

continued on next page

continued from previous page

	dy/dx	95% CI
Eastern	0.235	0.009, 0.461
Luapula	0.381	0.224, 0.537
Lusaka	0.125	-0.070, 0.319
Northern	0.192	0.014, 0.370
Northwestern	0.087	-0.134, 0.308
Southern	0.119	-0.073, 0.312
Western	1.269	1.066, 1.472
Religion		
Protestant	0.022	-0.007, 0.051
Muslim	0.129	-0.076, 0.334
Ethnicity		
Lunda (L)	0.117	-0.068, 0.303
Lala	−0.056	-0.137, 0.025
Ushi	0.045	-0.029, 0.119
Lamba	−0.009	-0.080, 0.063
Tonga	0.040	-0.016, 0.097
Luvale	−0.066	-0.136, 0.004
Lunda (NW)	−0.074	-0.151, 0.003
Mbunda	−0.010	-0.108, 0.088
Kaonde	−0.014	-0.082, 0.054
Lozi	−0.019	-0.083, 0.044
Chewa	−0.034	-0.092, 0.024
Nsenga	−0.036	-0.092, 0.019
Ngoni	−0.005	-0.076, 0.066
Mambwe	0.013	-0.084, 0.110
Namwanga	0.067	0.004, 0.130
Tumbuka	0.044	-0.029, 0.117
Other	0.012	-0.034, 0.058
Knows someone who died of AIDS	0.014	-0.006, 0.035
Would care for relative with AIDS	0.026	-0.018, 0.069
Age at first sex		
≤15	0.050	0.004, 0.096
>15	0.006	-0.040, 0.053
Married	−0.038	-0.088, 0.013
Drinks alcohol	0.007	-0.028, 0.043
Ever tested for HIV	0.010	-0.012, 0.032
Number of partners in last 12 months		
One	0.055	-0.009, 0.119
Multiple	0.142	0.009, 0.274
STD in last 12 months	0.072	0.024, 0.121
Smokes tobacco	0.154	0.021, 0.288
High risk sex in last 12 months	−0.038	-0.093, 0.017
Condom use at last sex	0.024	-0.010, 0.059
Interview language		
Bemba	0.040	-0.013, 0.093

continued on next page

continued from previous page

	dy/dx	95% CI
Lozi	0.118	-0.025, 0.262
Nyanja	0.103	0.035, 0.171
Tonga	0.053	-0.023, 0.130
Other	0.055	-0.049, 0.158
Interviewer 2	0.118	-0.016, 0.252
Interviewer 3	0.047	-0.093, 0.188
Interviewer 4	0.102	-0.039, 0.243
Interviewer 5	−0.060	-0.405, 0.284
Interviewer 6	−0.003	-0.342, 0.336
Interviewer 7	0.032	-0.309, 0.372
Interviewer 8	−0.009	-0.346, 0.329
Interviewer 9	−0.139	-0.470, 0.193
Interviewer 10	0.055	-0.231, 0.341
Interviewer 11	0.033	-0.311, 0.376
Interviewer 12	−0.024	-0.366, 0.318
Interviewer 13	−0.185	-0.390, 0.020
Interviewer 14	−0.093	-0.276, 0.090
Interviewer 15	−0.085	-0.276, 0.106
Interviewer 16	−0.043	-0.237, 0.151
Interviewer 17	−0.256	-0.349, -0.162
Interviewer 18	−0.169	-0.267, -0.070
Interviewer 19	−0.252	-0.349, -0.155
o.Interviewer 20	0.000	0.000, 0.000
Interviewer 21	0.120	-0.037, 0.277
Interviewer 22	0.131	0.002, 0.260
Interviewer 23	0.120	-0.005, 0.246
Interviewer 24	−0.047	-0.170, 0.076
Interviewer 25	0.169	-0.022, 0.361
Interviewer 26	0.046	-0.079, 0.170
Interviewer 27	−0.111	-0.234, 0.012
Interviewer 28	0.069	-0.095, 0.233
Interviewer 29	0.130	-0.102, 0.362
Interviewer 30	0.202	0.014, 0.390
Interviewer 31	0.113	-0.048, 0.274
Interviewer 32	−0.071	-0.204, 0.063
Interviewer 33	−0.196	-0.336, -0.057
Interviewer 34	−0.154	-0.295, -0.012
Interviewer 35	−0.104	-0.246, 0.037
Interviewer 36	0.070	-0.092, 0.233
Interviewer 37	0.089	-0.062, 0.239
Interviewer 38	0.067	-0.082, 0.215
Interviewer 39	−0.042	-0.176, 0.092
Interviewer 40	−0.044	-0.176, 0.088
Interviewer 41	0.023	-0.125, 0.170
Interviewer 42	−0.017	-0.144, 0.111

continued on next page

continued from previous page

	dy/dx	95% CI
Interviewer 43	−0.047	-0.201, 0.108
Interviewer 44	−1.172	-1.378, -0.966
Interviewer 45	−1.194	-1.397, -0.991
Interviewer 46	−1.132	-1.315, -0.950

N=6575, Censored cases=1325

ρ =-0.323, χ^2 =1.753, p=0.185

Table 3. Zambia 2007 Contact Regression Marginals, Male

	dy/dx	95% CI
Age category		
20 – 24	0.033	-0.014, 0.079
25 – 29	0.125	0.049, 0.202
30 – 34	0.188	0.085, 0.291
35 – 39	0.216	0.103, 0.330
40 – 44	0.226	0.110, 0.341
45 – 49	0.204	0.095, 0.313
50 – 54	0.156	0.067, 0.246
55 – 59	0.124	0.048, 0.199
Wealth quintile		
2nd	0.045	-0.003, 0.093
3rd	0.048	0.001, 0.095
4th	0.071	0.009, 0.133
5th	0.047	-0.013, 0.107
Mean education	0.002	-0.001, 0.005
Location		
Small city	0.003	-0.058, 0.064
Town	−0.022	-0.085, 0.040
Countryside	−0.063	-0.150, 0.025
Region		
Copperbelt	−0.042	-0.085, 0.002
Eastern	−0.028	-0.088, 0.033
Luapula	0.012	-0.029, 0.053
Lusaka	0.001	-0.058, 0.059
Northern	−0.075	-0.145, -0.005
Northwestern	−0.096	-0.171, -0.021
Southern	−0.001	-0.048, 0.045
Western	0.011	-0.036, 0.058
Age category		
20 – 24	−0.021	-0.056, 0.013
25 – 29	−0.039	-0.077, -0.001
30 – 34	−0.011	-0.048, 0.027
35 – 39	−0.011	-0.053, 0.031
40 – 44	0.025	-0.021, 0.071
45 – 49	0.005	-0.043, 0.053
50 – 54	0.007	-0.052, 0.066
55 – 59	0.039	-0.024, 0.101
Wealth quintile		
2nd	−0.054	-0.099, -0.009
3rd	−0.043	-0.090, 0.004
4th	−0.048	-0.107, 0.011
5th	−0.073	-0.143, -0.003

continued on next page

continued from previous page

	dy/dx	95% CI
Mean education	0.006	0.002, 0.011
Location		
Small city	0.149	0.012, 0.285
Town	0.160	0.036, 0.285
Countryside	0.201	0.076, 0.325
Region		
Copperbelt	−0.069	-0.213, 0.074
Eastern	0.224	0.065, 0.382
Luapula	0.064	-0.095, 0.223
Lusaka	0.225	0.043, 0.408
Northern	0.071	-0.103, 0.245
Northwestern	1.632	1.438, 1.826
Southern	0.158	0.005, 0.310
Western	0.114	-0.061, 0.288
Interviewer 2	0.033	-0.071, 0.136
Interviewer 3	0.021	-0.109, 0.150
Interviewer 4	0.069	-0.018, 0.155
Interviewer 5	0.053	-0.100, 0.205
Interviewer 6	0.140	0.019, 0.261
Interviewer 7	−0.050	-0.194, 0.093
Interviewer 8	−0.059	-0.191, 0.072
Interviewer 9	0.106	-0.007, 0.219
Interviewer 10	0.003	-0.123, 0.130
Interviewer 11	0.152	0.029, 0.276
Interviewer 12	0.065	-0.074, 0.203
Interviewer 13	0.029	-0.111, 0.169
Interviewer 14	−0.113	-0.260, 0.033
Interviewer 15	−0.144	-0.239, -0.050
Interviewer 16	0.020	-0.100, 0.139
Interviewer 17	−0.084	-0.225, 0.058
Interviewer 18	−0.128	-0.262, 0.007
o.Interviewer 19	0.000	0.000, 0.000
Interviewer 20	0.065	-0.070, 0.200
Interviewer 21	−0.011	-0.149, 0.128
Interviewer 22	−0.012	-0.216, 0.192
Interviewer 23	0.003	-0.140, 0.146
Interviewer 24	0.051	-0.120, 0.221
Interviewer 25	−0.116	-0.254, 0.021
Interviewer 26	0.092	-0.040, 0.224
Interviewer 27	0.006	-0.151, 0.164
Interviewer 28	0.141	-0.018, 0.300
Interviewer 29	0.046	-0.118, 0.210
Interviewer 30	−0.034	-0.154, 0.086
Interviewer 31	−0.064	-0.190, 0.061
Interviewer 32	−0.230	-0.367, -0.093

continued on next page

continued from previous page

	dy/dx	95% CI
Interviewer 33	0.131	0.019, 0.244
Interviewer 34	0.196	0.071, 0.321
Interviewer 35	0.123	-0.123, 0.368
Interviewer 36	0.170	0.000, 0.339
Interviewer 37	−0.014	-0.184, 0.156
Interviewer 38	0.027	-0.198, 0.252
Interviewer 39	−0.095	-0.292, 0.101
Interviewer 40	−0.053	-0.291, 0.184
Interviewer 41	−0.178	-0.384, 0.028
Interviewer 42	−0.080	-0.263, 0.103
Interviewer 43	−1.517	-1.697, -1.336
Interviewer 44	−1.565	-1.715, -1.414
Interviewer 45	−1.540	-1.725, -1.355
Interviewer 46	−1.601	-1.766, -1.436
Interviewer 47	−1.612	-1.779, -1.444
Interviewer 48	−1.605	-1.767, -1.443
Interviewer 49	−0.001	-0.123, 0.121
Interviewer 50	−0.163	-0.280, -0.047
Interviewer 51	−0.091	-0.216, 0.034
Interviewer 52	0.075	-0.078, 0.228
Interviewer 53	0.013	-0.136, 0.161
Interviewer 54	0.021	-0.129, 0.172
First day	0.024	-0.004, 0.051

N=7116, Censored cases=1971

ρ =-0.146, χ^2 =0.113, p=0.737

Table 4. Zambia 2007 Consent Regression Marginals, Male

	dy/dx	95% CI
Age category		
20 – 24	0.046	-0.010, 0.102
25 – 29	0.165	0.108, 0.223
30 – 34	0.254	0.192, 0.315
35 – 39	0.280	0.217, 0.343
40 – 44	0.302	0.234, 0.370
45 – 49	0.282	0.209, 0.354
50 – 54	0.221	0.150, 0.291
55 – 59	0.172	0.097, 0.247
Wealth quintile		
2nd	0.056	0.015, 0.096
3rd	0.058	0.016, 0.100
4th	0.076	0.028, 0.124
5th	0.051	-0.005, 0.107
Mean education	0.000	-0.004, 0.004
Location		
Small city	−0.060	-0.148, 0.029
Town	−0.079	-0.156, -0.002
Countryside	−0.132	-0.213, -0.051
Region		
Copperbelt	−0.014	-0.078, 0.051
Eastern	−0.013	-0.080, 0.054
Luapula	−0.011	-0.082, 0.060
Lusaka	−0.022	-0.093, 0.049
Northern	−0.087	-0.154, -0.020
Northwestern	−0.014	-0.132, 0.104
Southern	0.034	-0.048, 0.116
Western	−0.133	-0.257, -0.010
Religion		
Protestant	0.027	-0.001, 0.055
Muslim	−0.046	-0.224, 0.132
Ethnicity		
Lunda (L)	0.029	-0.046, 0.104
Lala	−0.026	-0.106, 0.053
Ushi	0.052	-0.033, 0.136
Lamba	−0.076	-0.204, 0.051
Tonga	0.012	-0.045, 0.068
Luvale	0.017	-0.063, 0.097
Lunda (NW)	−0.053	-0.147, 0.042
Mbunda	−0.043	-0.133, 0.047
Kaonde	−0.021	-0.100, 0.059
Lozi	0.034	-0.026, 0.093

continued on next page

continued from previous page

	dy/dx	95% CI
Chewa	−0.006	-0.054, 0.042
Nsenga	0.035	-0.020, 0.091
Ngoni	−0.032	-0.090, 0.025
Mambwe	−0.053	-0.131, 0.025
Namwanga	−0.069	-0.153, 0.015
Tumbuka	−0.097	-0.173, -0.022
Other	−0.005	-0.042, 0.032
Knows someone who died of AIDS	−0.012	-0.039, 0.014
Would care for relative with AIDS	0.018	-0.049, 0.085
Age at first sex		
≤15	−0.018	-0.086, 0.049
>15	−0.018	-0.084, 0.047
Married	−0.041	-0.089, 0.007
Drinks alcohol	0.022	-0.003, 0.047
Ever tested for HIV	0.037	0.012, 0.062
Number of partners last 12 months		
Single	0.046	-0.016, 0.107
Multiple	0.113	0.031, 0.196
STD in last 12 months	0.117	0.073, 0.160
Smokes tobacco	−0.023	-0.055, 0.009
High risk sex in last 12 months	−0.040	-0.090, 0.010
Condom use at last sex	0.063	0.032, 0.094
Interview language		
Bemba	0.021	-0.039, 0.080
Lozi	0.136	0.021, 0.251
Nyanja	−0.009	-0.073, 0.055
Tonga	−0.058	-0.135, 0.020
Other	−0.077	-0.191, 0.036
Age category		
20 – 24	−0.042	-0.077, -0.007
25 – 29	−0.051	-0.096, -0.005
30 – 34	−0.031	-0.076, 0.014
35 – 39	−0.016	-0.066, 0.035
40 – 44	0.009	-0.044, 0.061
45 – 49	−0.027	-0.083, 0.029
50 – 54	−0.012	-0.083, 0.060
55 – 59	0.036	-0.032, 0.103
Wealth quintile		
2nd	−0.017	-0.057, 0.023
3rd	−0.023	-0.065, 0.019
4th	0.007	-0.042, 0.056
5th	0.008	-0.052, 0.069
Mean education	0.005	0.001, 0.009
Location		
Small city	0.149	0.026, 0.272

continued on next page

continued from previous page

	dy/dx	95% CI
Town	0.151	0.040, 0.262
Countryside	0.186	0.075, 0.297
Region		
Copperbelt	0.006	-0.119, 0.132
Eastern	−0.041	-0.177, 0.094
Luapula	−0.007	-0.320, 0.305
Lusaka	0.128	-0.020, 0.276
Northern	0.052	-0.056, 0.159
Northwestern	0.157	0.008, 0.306
Southern	−0.003	-0.141, 0.135
Western	0.123	-0.051, 0.298
Religion		
Protestant	0.008	-0.018, 0.034
Muslim	0.099	-0.079, 0.277
Ethnicity		
Lunda (L)	0.035	-0.070, 0.140
Lala	−0.059	-0.135, 0.018
Ushi	−0.078	-0.158, 0.001
Lamba	−0.034	-0.110, 0.042
Tonga	0.017	-0.035, 0.068
Luvale	−0.006	-0.082, 0.070
Lunda (NW)	−0.034	-0.123, 0.054
Mbunda	0.013	-0.096, 0.122
Kaonde	0.062	-0.017, 0.141
Lozi	0.019	-0.043, 0.080
Chewa	−0.009	-0.061, 0.044
Nsenga	−0.017	-0.076, 0.043
Ngoni	0.045	-0.014, 0.105
Mambwe	0.011	-0.084, 0.106
Namwanga	0.032	-0.030, 0.094
Tumbuka	0.135	0.070, 0.200
Other	−0.025	-0.066, 0.017
Knows someone who died of AIDS	0.037	0.016, 0.057
Would care for relative with AIDS	0.044	0.001, 0.087
Age at first sex		
≤15	0.050	0.005, 0.094
>15	0.038	-0.007, 0.083
Married	0.019	-0.030, 0.068
Drinks alcohol	0.009	-0.015, 0.034
Ever tested for HIV	0.005	-0.021, 0.032
Number of partners last 12 months		
Single	−0.051	-0.109, 0.007
Multiple	−0.032	-0.119, 0.054
STD in last 12 months	0.042	-0.007, 0.092
Smokes tobacco	0.037	0.011, 0.062

continued on next page

continued from previous page

	dy/dx	95% CI
High risk sex in last 12 months	0.053	0.001, 0.106
Condom use at last sex	−0.006	-0.037, 0.025
Interview language		
Bemba	0.057	0.002, 0.111
Lozi	0.027	-0.103, 0.156
Nyanja	0.121	0.062, 0.181
Tonga	0.102	0.024, 0.180
Other	−0.005	-0.145, 0.134
Interviewer 2	0.003	-0.085, 0.092
Interviewer 3	0.039	-0.052, 0.130
Interviewer 4	0.065	-0.033, 0.163
Interviewer 5	−0.028	-0.101, 0.046
Interviewer 6	−0.084	-0.206, 0.039
Interviewer 7	−0.042	-0.137, 0.054
Interviewer 8	−0.084	-0.179, 0.011
Interviewer 9	0.023	-0.064, 0.110
Interviewer 10	−0.000	-0.104, 0.103
Interviewer 11	0.164	0.062, 0.265
Interviewer 12	0.078	-0.060, 0.216
Interviewer 13	0.153	-0.156, 0.461
Interviewer 14	0.084	-0.208, 0.376
Interviewer 15	0.074	-0.035, 0.183
Interviewer 16	−0.100	-0.206, 0.005
Interviewer 17	0.027	-0.077, 0.130
Interviewer 18	−0.116	-0.198, -0.033
Interviewer 19	−0.021	-0.090, 0.047
Interviewer 20	0.162	0.031, 0.293
Interviewer 21	0.062	-0.043, 0.168
Interviewer 22	−0.048	-0.155, 0.059
Interviewer 23	−0.087	-0.179, 0.006
Interviewer 24	−0.028	-0.185, 0.129
Interviewer 25	0.073	-0.105, 0.250
Interviewer 26	−0.080	-0.176, 0.017
Interviewer 27	−0.075	-0.185, 0.034
Interviewer 28	−0.070	-0.170, 0.031
Interviewer 29	0.079	-0.045, 0.203
Interviewer 30	−0.084	-0.195, 0.027
Interviewer 31	−0.021	-0.144, 0.102
Interviewer 32	−0.016	-0.121, 0.089
Interviewer 33	−0.034	-0.171, 0.104
Interviewer 34	−0.021	-0.162, 0.120

N=6372, Censored cases=1302

ρ =-0.717, χ^2 =6.109, p=0.013

Table 5. Swaziland 2007 Contact Regression Marginals, Female

	dy/dx	95% CI
Age category		
20 – 24	0.341	0.247, 0.436
25 – 29	0.440	0.319, 0.561
30 – 34	0.396	0.296, 0.496
35 – 39	0.328	0.233, 0.424
40 – 44	0.229	0.136, 0.323
45 – 49	0.159	0.088, 0.230
Wealth quintile		
2nd	0.005	-0.048, 0.058
3rd	0.009	-0.046, 0.064
4th	0.005	-0.049, 0.059
5th	−0.053	-0.126, 0.021
Mean education	−0.014	-0.018, -0.010
Town	0.063	-0.002, 0.127
Countryside	−0.060	-0.154, 0.033
Region		
Manzini	−0.016	-0.061, 0.028
Shiselweni	−0.015	-0.062, 0.032
Lubombo	−0.024	-0.073, 0.024
Age category		
20 – 24	−0.029	-0.055, -0.002
25 – 29	−0.040	-0.067, -0.014
30 – 34	−0.021	-0.048, 0.006
35 – 39	−0.017	-0.048, 0.015
40 – 44	−0.037	-0.067, -0.007
45 – 49	0.003	-0.036, 0.042
Wealth quintile		
2nd	−0.018	-0.055, 0.019
3rd	−0.016	-0.054, 0.021
4th	−0.014	-0.051, 0.023
5th	−0.071	-0.109, -0.032
Mean education	−0.005	-0.008, -0.002
Town	0.030	-0.008, 0.068
Countryside	0.067	0.033, 0.100
Region		
Manzini	0.001	-0.037, 0.040
Shiselweni	−0.002	-0.036, 0.033
Lubombo	−0.011	-0.048, 0.025
Interviewer 2	0.024	-0.102, 0.151
Interviewer 3	0.083	-0.045, 0.211
Interviewer 4	0.063	-0.052, 0.177
Interviewer 5	0.165	0.045, 0.284

continued on next page

continued from previous page

	dy/dx	95% CI
Interviewer 6	0.047	-0.054, 0.148
Interviewer 7	0.073	-0.032, 0.179
Interviewer 8	−0.031	-0.140, 0.077
Interviewer 9	0.063	-0.045, 0.171
Interviewer 10	0.090	-0.034, 0.214
Interviewer 11	−0.022	-0.139, 0.094
Interviewer 12	0.037	-0.075, 0.149
Interviewer 13	0.035	-0.076, 0.147
Interviewer 14	−0.029	-0.142, 0.083
Interviewer 15	0.020	-0.081, 0.120
Interviewer 16	−0.060	-0.180, 0.059
Interviewer 17	0.065	-0.062, 0.192
Interviewer 18	0.023	-0.076, 0.123
Interviewer 19	0.063	-0.050, 0.175
Interviewer 20	0.012	-0.092, 0.115
Interviewer 21	0.033	-0.076, 0.142
Interviewer 22	0.019	-0.103, 0.142
Interviewer 23	0.115	-0.000, 0.230
Interviewer 24	0.065	-0.043, 0.173
Interviewer 25	0.009	-0.104, 0.121
Interviewer 26	−0.002	-0.123, 0.118
Interviewer 27	0.039	-0.064, 0.141
Interviewer 28	−0.040	-0.143, 0.063
Interviewer 29	0.029	-0.099, 0.157
Interviewer 30	0.034	-0.074, 0.143
Interviewer 31	0.225	0.060, 0.390
Interviewer 32	0.084	-0.037, 0.205
Interviewer 33	−0.000	-0.103, 0.102
Interviewer 34	0.005	-0.105, 0.115
Interviewer 35	0.086	-0.021, 0.193
Interviewer 36	0.017	-0.101, 0.135
Interviewer 37	0.088	-0.039, 0.215
Interviewer 38	0.110	-0.034, 0.254
Interviewer 39	−0.020	-0.140, 0.100
Interviewer 40	0.012	-0.105, 0.128
Interviewer 41	0.105	-0.006, 0.216
Interviewer 42	0.110	-0.014, 0.233
Interviewer 43	0.019	-0.090, 0.127
Interviewer 44	0.060	-0.051, 0.171
Interviewer 45	0.102	-0.006, 0.209
Interviewer 46	0.095	-0.012, 0.202
Interviewer 47	0.008	-0.082, 0.098
Interviewer 48	0.061	-0.058, 0.179
Interviewer 49	0.076	-0.033, 0.184
Interviewer 50	0.066	-0.048, 0.179

continued on next page

continued from previous page

	dy/dx	95% CI
Interviewer 51	0.016	-0.083, 0.115
Interviewer 52	−0.003	-0.111, 0.104
Interviewer 53	−0.018	-0.127, 0.090
First day	0.007	-0.014, 0.028

N=5265, Censored cases=666
ρ =0.045, χ^2 =0.004, p=0.949

Table 6. Swaziland 2007 Consent Regression Marginals, Female

	dy/dx	95% CI
Age category		
20 – 24	0.255	0.197, 0.313
25 – 29	0.377	0.308, 0.446
30 – 34	0.359	0.290, 0.428
35 – 39	0.298	0.227, 0.369
40 – 44	0.218	0.136, 0.301
45 – 49	0.103	0.014, 0.192
Wealth quintile		
2nd	0.005	-0.055, 0.064
3rd	0.019	-0.041, 0.079
4th	0.020	-0.044, 0.083
5th	−0.014	-0.095, 0.067
Mean education	−0.011	-0.018, -0.003
Town	0.054	-0.022, 0.130
Countryside	−0.044	-0.131, 0.043
Region		
Manzini	−0.055	-0.103, -0.007
Shiselweni	−0.051	-0.101, -0.000
Lubombo	−0.046	-0.097, 0.004
Religion		
Protestant	0.028	-0.028, 0.084
Roman Catholic	0.026	-0.057, 0.109
Pentecostal	0.029	-0.063, 0.121
Zionist	0.055	0.000, 0.110
Apostolic sect	0.071	-0.003, 0.146
Other	0.026	-0.228, 0.280
None	0.030	-0.060, 0.119
Knows someone who died of AIDS	0.020	-0.022, 0.062
Would care for relative with AIDS	0.039	-0.036, 0.115
Age at first sex		
≤15 yrs	0.231	0.135, 0.326
>15 yrs	0.233	0.147, 0.320
Married	−0.131	-0.191, -0.072
Ever tested for HIV	0.039	0.005, 0.072
Number of partners in last 12 months		
One	0.042	-0.033, 0.117
Multiple	0.144	0.004, 0.284
STD in last 12 months	0.115	0.060, 0.169
Smokes tobacco	0.198	0.065, 0.330
High risk sex in last 12 months	−0.001	-0.058, 0.056
Condom use at last sex	0.105	0.064, 0.146
Age category		

continued on next page

continued from previous page

	dy/dx	95% CI
20 – 24	−0.030	-0.064, 0.003
25 – 29	−0.033	-0.070, 0.003
30 – 34	−0.042	-0.079, -0.004
35 – 39	−0.023	-0.059, 0.012
40 – 44	−0.039	-0.070, -0.007
45 – 49	−0.029	-0.074, 0.016
Wealth quintile		
2nd	−0.017	-0.051, 0.018
3rd	−0.015	-0.049, 0.019
4th	−0.016	-0.048, 0.016
5th	−0.035	-0.066, -0.003
Mean education	−0.005	-0.008, -0.003
Town	0.004	-0.027, 0.034
Countryside	0.043	0.017, 0.068
Region		
Manzini	0.008	-0.019, 0.036
Shiselweni	−0.009	-0.040, 0.021
Lubombo	−0.001	-0.031, 0.029
Religion		
Protestant	0.001	-0.018, 0.021
Roman Catholic	−0.021	-0.053, 0.012
Pentecostal	−0.012	-0.045, 0.021
Zionist	−0.003	-0.023, 0.017
Apostolic sect	0.012	-0.021, 0.045
Other	−0.112	-0.156, -0.069
None	0.004	-0.037, 0.046
Knows someone who died of AIDS	0.024	0.008, 0.040
Would care for relative with AIDS	0.017	-0.015, 0.049
Age at first sex		
≤15 yrs	0.006	-0.026, 0.038
>15 yrs	0.007	-0.026, 0.040
Married	−0.009	-0.037, 0.019
Ever tested for HIV	−0.005	-0.020, 0.011
Number of partners in last 12 months		
One	0.017	-0.014, 0.048
Multiple	−0.008	-0.069, 0.052
STD in last 12 months	0.026	-0.003, 0.055
Smokes tobacco	−0.002	-0.058, 0.055
High risk sex in last 12 months	0.008	-0.020, 0.037
Condom use at last sex	−0.000	-0.017, 0.017
Interviewer 2	0.025	-0.044, 0.095
Interviewer 3	0.009	-0.064, 0.081
Interviewer 4	0.008	-0.066, 0.082
Interviewer 5	−0.027	-0.102, 0.049
Interviewer 6	−0.015	-0.103, 0.073

continued on next page

continued from previous page

	dy/dx	95% CI
Interviewer 7	0.036	-0.043, 0.116
Interviewer 8	−0.003	-0.119, 0.112
Interviewer 9	−0.022	-0.094, 0.050
Interviewer 10	−0.027	-0.105, 0.051
Interviewer 11	−0.035	-0.105, 0.035
Interviewer 12	−0.010	-0.087, 0.067
Interviewer 13	−0.005	-0.089, 0.080
Interviewer 14	0.003	-0.070, 0.075
Interviewer 15	−0.008	-0.081, 0.064
Interviewer 16	−0.040	-0.122, 0.043
Interviewer 17	−0.037	-0.115, 0.042
Interviewer 18	−0.019	-0.108, 0.070
Interviewer 19	0.073	-0.020, 0.166
Interviewer 20	0.027	-0.045, 0.099
Interviewer 21	0.016	-0.061, 0.093
Interviewer 22	−0.001	-0.079, 0.078
Interviewer 23	−0.010	-0.092, 0.073
Interviewer 24	0.014	-0.067, 0.095
Interviewer 25	0.005	-0.070, 0.080
Interviewer 26	0.019	-0.055, 0.092
Interviewer 27	0.013	-0.067, 0.092
Interviewer 28	−0.020	-0.111, 0.071
Interviewer 29	−0.003	-0.071, 0.065
Interviewer 30	0.023	-0.049, 0.094
Interviewer 31	−0.034	-0.130, 0.062
Interviewer 32	−0.007	-0.080, 0.065
Interviewer 33	−0.028	-0.094, 0.038

N=3851, Censored cases=293

ρ =-0.536, χ^2 =0.504, p=0.478

Table 7. Swaziland 2007 Contact Regression Marginals, Male

	dy/dx	95% CI
Age category		
20 – 24	0.294	0.028, 0.559
25 – 29	0.432	0.115, 0.749
30 – 34	0.550	0.185, 0.915
35 – 39	0.561	0.194, 0.927
40 – 44	0.527	0.163, 0.890
45 – 49	0.445	0.166, 0.724
Wealth quintile		
2nd	0.046	-0.039, 0.132
3rd	0.000	-0.056, 0.057
4th	−0.009	-0.072, 0.055
5th	−0.056	-0.121, 0.008
Mean education	−0.005	-0.009, -0.001
Town	0.041	-0.027, 0.109
Countryside	−0.038	-0.161, 0.085
Region		
Manzini	−0.015	-0.067, 0.038
Shiselweni	−0.025	-0.085, 0.034
Lubombo	−0.038	-0.101, 0.025
Age category		
20 – 24	−0.135	-0.170, -0.099
25 – 29	−0.138	-0.177, -0.099
30 – 34	−0.140	-0.186, -0.094
35 – 39	−0.134	-0.182, -0.087
40 – 44	−0.154	-0.203, -0.105
45 – 49	−0.101	-0.154, -0.048
Wealth quintile		
2nd	−0.041	-0.100, 0.018
3rd	−0.000	-0.058, 0.057
4th	−0.024	-0.080, 0.033
5th	−0.031	-0.091, 0.028
Mean education	−0.003	-0.007, 0.000
Town	0.049	-0.009, 0.106
Countryside	0.087	0.038, 0.135
Region		
Manzini	−0.044	-0.101, 0.013
Shiselweni	−0.043	-0.100, 0.013
Lubombo	−0.003	-0.057, 0.051
Interviewer 2	0.118	-0.070, 0.306
Interviewer 3	0.168	-0.039, 0.375
Interviewer 4	0.149	0.000, 0.298
Interviewer 5	0.167	0.012, 0.321

continued on next page

continued from previous page

	dy/dx	95% CI
Interviewer 6	0.050	-0.132, 0.231
Interviewer 7	0.062	-0.132, 0.257
Interviewer 8	−0.039	-0.179, 0.101
Interviewer 9	0.016	-0.189, 0.220
Interviewer 10	0.107	-0.125, 0.339
Interviewer 11	−0.004	-0.159, 0.151
Interviewer 12	0.079	-0.092, 0.251
Interviewer 13	0.156	-0.084, 0.397
Interviewer 14	0.052	-0.158, 0.262
Interviewer 15	0.040	-0.135, 0.215
Interviewer 16	0.056	-0.097, 0.210
Interviewer 17	−0.025	-0.216, 0.167
Interviewer 18	0.014	-0.140, 0.169
Interviewer 19	0.086	-0.095, 0.266
Interviewer 20	0.037	-0.119, 0.193
Interviewer 21	−0.047	-0.192, 0.097
Interviewer 22	0.055	-0.085, 0.195
Interviewer 23	0.158	-0.018, 0.333
Interviewer 24	0.161	-0.063, 0.385
Interviewer 25	0.096	-0.098, 0.291
Interviewer 26	0.112	-0.101, 0.325
Interviewer 27	0.023	-0.170, 0.216
Interviewer 28	−0.013	-0.214, 0.187
Interviewer 29	0.116	-0.066, 0.299
Interviewer 30	0.140	-0.043, 0.322
Interviewer 31	0.025	-0.109, 0.160
Interviewer 32	0.130	-0.055, 0.315
Interviewer 33	0.043	-0.119, 0.205
Interviewer 34	0.061	-0.084, 0.206
Interviewer 35	0.022	-0.192, 0.236
Interviewer 36	0.024	-0.175, 0.222
Interviewer 37	−0.006	-0.193, 0.181
Interviewer 38	0.211	-0.047, 0.469
Interviewer 39	0.097	-0.110, 0.304
Interviewer 40	0.156	-0.118, 0.430
Interviewer 41	0.006	-0.204, 0.217
Interviewer 42	0.143	-0.031, 0.318
Interviewer 43	0.020	-0.214, 0.255
Interviewer 44	0.081	-0.094, 0.257
Interviewer 45	0.090	-0.086, 0.265
Interviewer 46	0.112	-0.072, 0.295
Interviewer 47	0.103	-0.038, 0.244
Interviewer 48	0.017	-0.149, 0.184
Interviewer 49	0.040	-0.136, 0.216
Interviewer 50	0.100	-0.051, 0.251

continued on next page

continued from previous page

	dy/dx	95% CI
Interviewer 51	0.139	-0.078, 0.356
Interviewer 52	−0.012	-0.156, 0.131
Interviewer 53	−0.024	-0.184, 0.136
First day	0.011	-0.020, 0.042

N=4647, Censored cases=1032

ρ =-0.490, χ^2 =0.200, p=0.655

Table 8. Swaziland 2007 Consent Regression Marginals, Male

	dy/dx	95% CI
Age category		
20 – 24	0.175	0.067, 0.284
25 – 29	0.281	0.166, 0.396
30 – 34	0.400	0.269, 0.531
35 – 39	0.437	0.298, 0.576
40 – 44	0.408	0.268, 0.547
45 – 49	0.324	0.219, 0.430
Wealth quintile		
2nd	0.041	-0.021, 0.103
3rd	0.004	-0.054, 0.061
4th	0.012	-0.055, 0.078
5th	−0.025	-0.091, 0.042
Mean education	−0.004	-0.008, 0.000
Town	0.051	-0.006, 0.108
Countryside	−0.017	-0.080, 0.045
Region		
Manzini	−0.032	-0.073, 0.009
Shiselweni	−0.035	-0.086, 0.016
Lubombo	−0.051	-0.096, -0.005
Religion		
Protestant	−0.040	-0.108, 0.029
Roman Catholic	0.002	-0.079, 0.084
Pentecostal	−0.092	-0.194, 0.010
Zionist	0.019	-0.043, 0.081
Apostolic sect	0.024	-0.062, 0.110
Other	−0.116	-0.238, 0.005
None	−0.003	-0.066, 0.061
Knows someone who died of AIDS	−0.027	-0.064, 0.009
Would care for relative with AIDS	0.005	-0.059, 0.069
Age at first sex		
≤15 yrs	0.019	-0.049, 0.087
>15 yrs	0.054	-0.006, 0.114
Married	−0.070	-0.122, -0.019
Ever tested for HIV	0.060	0.023, 0.098
Number of partners in last 12 months		
One	0.098	0.037, 0.158
Multiple	0.186	0.111, 0.260
STD in last 12 months	0.118	0.074, 0.162
Smokes tobacco	0.019	-0.018, 0.057
High risk sex in last 12 months	−0.045	-0.090, -0.000
Condom use at last sex	0.043	0.001, 0.086
Age category		

continued on next page

continued from previous page

	dy/dx	95% CI
20 – 24	−0.114	-0.150, -0.078
25 – 29	−0.112	-0.158, -0.066
30 – 34	−0.121	-0.170, -0.073
35 – 39	−0.115	-0.169, -0.062
40 – 44	−0.134	-0.190, -0.078
45 – 49	−0.080	-0.146, -0.015
Wealth quintile		
2nd	−0.055	-0.098, -0.011
3rd	−0.050	-0.091, -0.009
4th	−0.070	-0.112, -0.028
5th	−0.063	-0.112, -0.014
Mean education	−0.003	-0.006, 0.000
Town	−0.005	-0.047, 0.036
Countryside	0.043	0.006, 0.080
Region		
Manzini	0.003	-0.036, 0.041
Shiselweni	−0.027	-0.073, 0.019
Lubombo	−0.010	-0.046, 0.027
Religion		
Protestant	0.042	0.003, 0.080
Roman Catholic	−0.004	-0.060, 0.052
Pentecostal	0.015	-0.046, 0.077
Zionist	0.019	-0.017, 0.056
Apostolic sect	0.043	-0.006, 0.093
Other	0.008	-0.079, 0.095
None	0.011	-0.028, 0.049
Knows someone who died of AIDS	0.042	0.019, 0.065
Would care for relative with AIDS	0.007	-0.036, 0.051
Age at first sex		
≤15 yrs	−0.002	-0.048, 0.044
>15 yrs	0.023	-0.020, 0.067
Married	−0.046	-0.087, -0.006
Ever tested for HIV	−0.012	-0.038, 0.014
Number of partners in last 12 months		
One	0.017	-0.034, 0.068
Multiple	0.018	-0.046, 0.082
STD in last 12 months	0.074	0.030, 0.117
Smokes tobacco	−0.006	-0.036, 0.024
High risk sex in last 12 months	−0.015	-0.054, 0.024
Condom use at last sex	−0.042	-0.066, -0.018
Interviewer 2	−0.003	-0.091, 0.085
Interviewer 3	−0.029	-0.127, 0.070
Interviewer 4	−0.108	-0.207, -0.009
Interviewer 5	−0.103	-0.187, -0.019
Interviewer 6	−0.043	-0.127, 0.041

continued on next page

continued from previous page

	dy/dx	95% CI
Interviewer 7	−0.037	-0.125, 0.051
Interviewer 8	−0.031	-0.116, 0.053
Interviewer 9	−0.051	-0.129, 0.028
Interviewer 10	−0.045	-0.154, 0.063
Interviewer 11	−0.101	-0.208, 0.007
Interviewer 12	−0.123	-0.217, -0.029
Interviewer 13	−0.113	-0.207, -0.019
Interviewer 14	−0.038	-0.120, 0.045
Interviewer 15	−0.093	-0.169, -0.017
Interviewer 16	−0.100	-0.196, -0.004
Interviewer 17	−0.077	-0.177, 0.022
Interviewer 18	−0.012	-0.141, 0.117
Interviewer 19	−0.091	-0.201, 0.020
Interviewer 20	−0.004	-0.097, 0.090
Interviewer 21	−0.033	-0.135, 0.068
Interviewer 22	0.010	-0.115, 0.135
Interviewer 23	−0.061	-0.139, 0.018
Interviewer 24	−0.017	-0.123, 0.088
Interviewer 25	−0.060	-0.143, 0.023
Interviewer 26	−0.077	-0.163, 0.009

N=3979, Censored cases=515

ρ =-0.589, χ^2 =1.047, p=0.306

Table 9. Zimbabwe 2005 Contact Regression Marginals, Female

	dy/dx	95% CI
Age category		
20 – 24	0.156	0.081, 0.230
25 – 29	0.257	0.149, 0.366
30 – 34	0.292	0.166, 0.418
35 – 39	0.297	0.178, 0.416
40 – 44	0.228	0.124, 0.332
45 – 49	0.158	0.081, 0.234
Wealth quintile		
2nd	0.034	0.004, 0.063
3rd	0.045	0.011, 0.079
4th	0.054	0.013, 0.094
5th	−0.026	-0.072, 0.021
Mean education	−0.005	-0.009, -0.000
Location		
Small city	−0.003	-0.043, 0.038
Town	−0.014	-0.064, 0.035
Countryside	−0.051	-0.122, 0.020
Region		
Mashonaland central	−0.008	-0.058, 0.042
Mashonaland east	0.021	-0.030, 0.071
Mashonaland west	0.024	-0.018, 0.066
Matebeleland north	0.051	0.006, 0.096
Matebeleland south	0.054	0.008, 0.100
Midlands	0.020	-0.055, 0.094
Masvingo	0.018	-0.027, 0.062
Age category		
20 – 24	−0.007	-0.028, 0.014
25 – 29	0.003	-0.020, 0.027
30 – 34	−0.005	-0.031, 0.021
35 – 39	0.008	-0.020, 0.035
40 – 44	−0.014	-0.044, 0.015
45 – 49	0.001	-0.031, 0.034
Wealth quintile		
2nd	0.008	-0.021, 0.037
3rd	0.012	-0.023, 0.047
4th	−0.001	-0.041, 0.039
5th	−0.030	-0.076, 0.016
Mean education	0.001	-0.002, 0.005
Location		
Small city	−0.022	-0.097, 0.052
Town	−0.117	-0.184, -0.050
Countryside	0.005	-0.061, 0.072

continued on next page

continued from previous page

	dy/dx	95% CI
Region		
Mashonaland central	−0.083	-0.161, -0.006
Mashonaland east	0.055	-0.048, 0.157
Mashonaland west	0.077	-0.001, 0.156
Matebeleland north	0.023	-0.094, 0.140
Matebeleland south	0.127	-0.018, 0.272
Midlands	0.075	-0.028, 0.178
Masvingo	0.050	-0.025, 0.126
Interviewer 2	−0.008	-0.085, 0.068
Interviewer 3	−0.066	-0.176, 0.045
Interviewer 4	−0.005	-0.095, 0.084
Interviewer 5	−0.026	-0.120, 0.068
Interviewer 6	0.055	-0.047, 0.157
Interviewer 7	0.068	-0.058, 0.195
Interviewer 8	−0.062	-0.167, 0.043
Interviewer 9	−0.030	-0.141, 0.081
Interviewer 10	0.019	-0.085, 0.123
Interviewer 11	0.039	-0.084, 0.161
Interviewer 12	−0.025	-0.135, 0.084
Interviewer 13	0.054	-0.067, 0.176
Interviewer 14	0.022	-0.108, 0.153
Interviewer 15	−0.013	-0.153, 0.128
Interviewer 16	−0.017	-0.122, 0.088
Interviewer 17	0.076	-0.047, 0.200
Interviewer 18	0.100	-0.083, 0.283
Interviewer 19	0.099	-0.085, 0.283
Interviewer 20	0.122	-0.015, 0.259
Interviewer 21	0.170	-0.047, 0.386
Interviewer 22	0.179	-0.027, 0.386
Interviewer 23	0.003	-0.132, 0.138
Interviewer 24	0.203	-0.010, 0.415
Interviewer 25	0.211	0.042, 0.379
Interviewer 26	0.157	-0.023, 0.338
Interviewer 27	0.130	-0.036, 0.297
Interviewer 28	0.032	-0.065, 0.129
Interviewer 29	0.064	-0.045, 0.174
Interviewer 30	−0.011	-0.116, 0.094
Interviewer 31	0.053	-0.061, 0.167
Interviewer 32	0.048	-0.058, 0.154
Interviewer 33	0.082	-0.023, 0.188
Interviewer 34	−0.130	-0.314, 0.053
Interviewer 35	−0.155	-0.328, 0.018
Interviewer 36	−0.092	-0.230, 0.047
Interviewer 37	−0.113	-0.263, 0.036
Interviewer 38	−0.156	-0.333, 0.021

continued on next page

continued from previous page

	dy/dx	95% CI
Interviewer 39	−0.113	-0.285, 0.059
Interviewer 40	−0.028	-0.170, 0.115
Interviewer 41	−0.096	-0.235, 0.043
Interviewer 42	−0.079	-0.209, 0.051
Interviewer 43	0.130	-0.015, 0.275
Interviewer 44	0.019	-0.108, 0.146
Interviewer 45	−0.072	-0.206, 0.061
Interviewer 46	−0.001	-0.124, 0.122
Interviewer 47	0.162	-0.010, 0.334
Interviewer 48	−0.007	-0.124, 0.110
Interviewer 49	0.100	-0.049, 0.249
Interviewer 50	−0.048	-0.202, 0.106
Interviewer 51	−0.061	-0.176, 0.055
Interviewer 52	−0.076	-0.208, 0.055
Interviewer 53	−0.053	-0.165, 0.058
Interviewer 54	−0.044	-0.113, 0.025
Interviewer 55	−0.038	-0.138, 0.063
Interviewer 56	0.064	-0.056, 0.184
Interviewer 57	0.015	-0.174, 0.203
Interviewer 58	0.088	-0.052, 0.228
Interviewer 59	−0.005	-0.157, 0.147
Interviewer 60	0.053	-0.104, 0.209
Interviewer 61	0.037	-0.096, 0.169
Interviewer 62	0.013	-0.085, 0.111
Interviewer 63	−0.050	-0.156, 0.055
Interviewer 64	−0.075	-0.181, 0.031
Interviewer 65	−0.089	-0.179, 0.001
Interviewer 66	−0.096	-0.206, 0.015
Interviewer 67	0.014	-0.097, 0.125
Interviewer 68	−0.092	-0.196, 0.013
Interviewer 69	−0.018	-0.128, 0.092
Interviewer 70	−0.068	-0.167, 0.030
Interviewer 71	−0.043	-0.149, 0.062
Interviewer 72	−0.151	-0.246, -0.055
Interviewer 73	−0.081	-0.170, 0.008
Interviewer 74	−0.047	-0.123, 0.029
Interviewer 75	0.058	-0.043, 0.159
Interviewer 76	−0.053	-0.168, 0.062
First day	0.010	-0.009, 0.030

N=8894, Censored cases=1411

ρ =0.263, χ^2 =0.104, p=0.747

Table 10. Zimbabwe 2005 Consent Regression Marginals, Female

	dy/dx	95% CI
Age category		
20 – 24	0.107	0.064, 0.151
25 – 29	0.218	0.162, 0.274
30 – 34	0.250	0.190, 0.309
35 – 39	0.243	0.187, 0.299
40 – 44	0.170	0.112, 0.227
45 – 49	0.077	0.023, 0.132
Wealth quintile		
2nd	0.038	0.007, 0.068
3rd	0.047	0.012, 0.082
4th	0.052	0.012, 0.092
5th	−0.015	-0.066, 0.036
Mean education	−0.004	-0.008, 0.000
Location		
Small city	−0.060	-0.147, 0.028
Town	−0.016	-0.066, 0.034
Countryside	−0.071	-0.128, -0.013
Region		
Mashonaland central	0.018	-0.035, 0.071
Mashonaland east	0.007	-0.044, 0.058
Mashonaland west	0.029	-0.019, 0.076
Matebeleland north	0.009	-0.074, 0.091
Matebeleland south	0.003	-0.081, 0.087
Midlands	0.005	-0.062, 0.072
Masvingo	0.015	-0.031, 0.060
Religion		
Roman Catholic	0.077	-0.000, 0.154
Protestant	0.090	0.014, 0.167
Pentecostal	0.081	0.002, 0.160
Apostolic sect	0.089	0.011, 0.168
None	0.114	0.030, 0.199
Other	0.092	0.009, 0.175
Knows someone who died of AIDS	−0.003	-0.022, 0.015
Would care for relative with AIDS	−0.000	-0.039, 0.039
Age at first sex		
≤15 yrs	0.281	0.209, 0.354
>15 yrs	0.263	0.200, 0.326
Married	−0.127	-0.162, -0.092
Ever tested for HIV	0.013	-0.011, 0.038
Number of partners in last 12 months		
One	−0.015	-0.072, 0.042
Multiple	0.058	-0.049, 0.165

continued on next page

continued from previous page

	dy/dx	95% CI
STD in last 12 months	0.121	0.089, 0.154
Smokes tobacco	−0.051	-0.249, 0.146
High risk sex in last 12 months	0.031	-0.019, 0.081
Condom use at last sex	0.060	0.014, 0.106
Interview language		
Ndebele	0.044	-0.030, 0.118
English	−0.022	-0.108, 0.063
Age category		
20 – 24	−0.013	-0.037, 0.011
25 – 29	−0.005	-0.034, 0.025
30 – 34	−0.011	-0.042, 0.021
35 – 39	−0.001	-0.035, 0.033
40 – 44	−0.026	-0.060, 0.008
45 – 49	−0.010	-0.049, 0.029
Wealth quintile		
2nd	0.012	-0.018, 0.042
3rd	0.005	-0.028, 0.038
4th	−0.002	-0.041, 0.036
5th	−0.038	-0.083, 0.007
Mean education	−0.002	-0.005, 0.002
Location		
Small city	−0.121	-0.246, 0.004
Town	−0.102	-0.169, -0.035
Countryside	0.022	-0.043, 0.088
Region		
Mashonaland central	−0.053	-0.117, 0.010
Mashonaland east	0.062	-0.059, 0.182
Mashonaland west	0.074	-0.039, 0.187
Matebeleland north	−0.100	-0.241, 0.040
Matebeleland south	−0.049	-0.213, 0.115
Midlands	0.065	-0.055, 0.185
Masvingo	0.066	-0.028, 0.160
Religion		
Roman Catholic	−0.006	-0.063, 0.051
Protestant	−0.020	-0.074, 0.034
Pentecostal	−0.034	-0.089, 0.021
Apostolic sect	−0.044	-0.097, 0.008
None	−0.041	-0.099, 0.017
Other	−0.046	-0.103, 0.010
Knows someone who died of AIDS	0.012	-0.004, 0.029
Would care for relative with AIDS	0.037	0.014, 0.060
Age at first sex		
$\leq$15 yrs	−0.022	-0.056, 0.013
$>$15 yrs	−0.004	-0.035, 0.027
Married	−0.049	-0.084, -0.014

continued on next page

continued from previous page

	dy/dx	95% CI
Ever tested for HIV	0.022	0.003, 0.041
Number of partners in last 12 months		
One	0.022	-0.030, 0.073
Multiple	0.022	-0.070, 0.114
STD in last 12 months	0.047	0.015, 0.078
Smokes tobacco	−0.007	-0.123, 0.108
High risk sex in last 12 months	−0.021	-0.065, 0.024
Condom use at last sex	−0.026	-0.062, 0.010
Interview language		
Ndebele	0.037	-0.026, 0.101
English	−0.099	-0.158, -0.040
Interviewer 2	−0.059	-0.125, 0.007
Interviewer 3	−0.071	-0.137, -0.005
Interviewer 4	0.094	-0.051, 0.239
Interviewer 5	0.015	-0.106, 0.135
Interviewer 6	0.060	-0.075, 0.196
Interviewer 7	−0.017	-0.138, 0.103
Interviewer 8	−0.013	-0.139, 0.113
Interviewer 9	−0.061	-0.147, 0.025
Interviewer 10	0.028	-0.123, 0.179
Interviewer 11	0.044	-0.110, 0.199
Interviewer 12	−0.004	-0.124, 0.117
Interviewer 13	0.078	-0.090, 0.246
Interviewer 14	0.149	0.005, 0.293
Interviewer 15	0.104	-0.066, 0.274
Interviewer 16	−0.111	-0.199, -0.023
Interviewer 17	−0.016	-0.126, 0.094
Interviewer 18	0.003	-0.088, 0.094
Interviewer 19	−0.073	-0.277, 0.132
Interviewer 20	−0.002	-0.169, 0.164
Interviewer 21	−0.024	-0.194, 0.146
Interviewer 22	−0.036	-0.176, 0.104
Interviewer 23	0.125	-0.010, 0.260
Interviewer 24	0.004	-0.131, 0.139
Interviewer 25	−0.124	-0.242, -0.007
Interviewer 26	−0.079	-0.194, 0.036
Interviewer 27	−0.063	-0.175, 0.050
Interviewer 28	−0.047	-0.151, 0.058
Interviewer 29	−0.112	-0.253, 0.030
Interviewer 30	−0.182	-0.311, -0.052
Interviewer 31	−0.097	-0.237, 0.044
Interviewer 32	−0.099	-0.168, -0.030
Interviewer 33	0.005	-0.138, 0.147
Interviewer 34	−0.003	-0.148, 0.142
Interviewer 35	−0.025	-0.167, 0.116

continued on next page

continued from previous page

	dy/dx	95% CI
Interviewer 36	−0.077	-0.169, 0.016
Interviewer 37	−0.125	-0.212, -0.037
Interviewer 38	−0.033	-0.124, 0.059
Interviewer 39	−0.137	-0.228, -0.046
Interviewer 40	−0.098	-0.195, 0.000
Interviewer 41	−0.123	-0.214, -0.032
Interviewer 42	−0.078	-0.176, 0.020
Interviewer 43	−0.064	-0.199, 0.071

N=8498, Censored cases=1325

ρ =-0.206, χ^2 =0.341, p=0.559

Table 11. Zimbabwe 2005 Contact Regression Marginals, Male

	dy/dx	95% CI
Age category		
20 – 24	0.048	0.008, 0.089
25 – 29	0.159	0.092, 0.227
30 – 34	0.249	0.157, 0.340
35 – 39	0.266	0.168, 0.365
40 – 44	0.267	0.174, 0.360
45 – 49	0.226	0.139, 0.313
50 – 54	0.186	0.109, 0.264
Wealth quintile		
2nd	0.003	-0.027, 0.033
3rd	0.001	-0.031, 0.033
4th	0.022	-0.020, 0.064
5th	−0.006	-0.056, 0.044
Mean education	−0.003	-0.007, 0.000
Location		
Small city	−0.024	-0.066, 0.018
Town	−0.012	-0.058, 0.034
Countryside	−0.025	-0.074, 0.024
Region		
Mashonaland central	−0.010	-0.064, 0.044
Mashonaland east	−0.002	-0.045, 0.041
Mashonaland west	0.003	-0.039, 0.045
Matebeleland north	−0.001	-0.050, 0.047
Matebeleland south	0.019	-0.030, 0.068
Midlands	−0.023	-0.072, 0.026
Masvingo	−0.006	-0.042, 0.031
Age category		
20 – 24	−0.030	-0.059, -0.001
25 – 29	−0.051	-0.081, -0.021
30 – 34	−0.054	-0.086, -0.021
35 – 39	−0.058	-0.095, -0.021
40 – 44	−0.025	-0.067, 0.018
45 – 49	−0.037	-0.081, 0.007
50 – 54	−0.047	-0.096, 0.002
Wealth quintile		
2nd	−0.004	-0.043, 0.036
3rd	−0.018	-0.058, 0.022
4th	−0.040	-0.083, 0.004
5th	−0.076	-0.131, -0.021
Mean education	−0.001	-0.006, 0.003
Location		
Small city	−0.096	-0.206, 0.014

continued on next page

continued from previous page

	dy/dx	95% CI
Town	−0.085	-0.161, -0.009
Countryside	0.062	-0.016, 0.140
Region		
Mashonaland central	−0.193	-0.276, -0.110
Mashonaland east	−0.020	-0.125, 0.085
Mashonaland west	0.044	-0.051, 0.140
Matebeleland north	−0.170	-0.308, -0.031
Matebeleland south	−0.171	-0.337, -0.005
Midlands	0.081	-0.033, 0.194
Masvingo	−0.027	-0.117, 0.062
Interviewer 2	−0.007	-0.136, 0.122
Interviewer 3	−0.029	-0.139, 0.082
Interviewer 4	0.029	-0.135, 0.194
Interviewer 5	0.140	0.016, 0.263
Interviewer 6	−0.004	-0.165, 0.156
Interviewer 7	0.008	-0.118, 0.135
Interviewer 8	0.129	-0.033, 0.290
Interviewer 9	0.172	0.029, 0.315
Interviewer 10	0.176	0.020, 0.331
Interviewer 11	0.074	-0.074, 0.221
Interviewer 12	0.203	0.019, 0.388
Interviewer 13	0.027	-0.147, 0.200
Interviewer 14	0.086	-0.097, 0.270
Interviewer 15	−0.007	-0.164, 0.149
Interviewer 16	0.154	0.003, 0.306
Interviewer 17	0.134	-0.052, 0.321
Interviewer 18	0.101	-0.091, 0.294
Interviewer 19	0.136	-0.030, 0.302
Interviewer 20	0.057	-0.126, 0.241
Interviewer 21	0.255	0.005, 0.504
Interviewer 22	0.144	-0.074, 0.361
Interviewer 23	0.154	-0.028, 0.335
Interviewer 24	0.160	0.001, 0.319
Interviewer 25	0.186	0.009, 0.362
Interviewer 26	0.130	-0.028, 0.288
Interviewer 27	−0.005	-0.131, 0.121
Interviewer 28	0.041	-0.097, 0.179
Interviewer 29	0.025	-0.108, 0.158
Interviewer 30	0.069	-0.076, 0.213
Interviewer 31	0.171	0.032, 0.309
Interviewer 32	0.183	0.053, 0.313
Interviewer 33	0.097	-0.141, 0.335
Interviewer 34	−0.042	-0.215, 0.130
Interviewer 35	0.238	0.014, 0.463
Interviewer 36	0.222	0.001, 0.442

continued on next page

continued from previous page

	dy/dx	95% CI
Interviewer 37	−0.018	-0.228, 0.193
Interviewer 38	0.038	-0.188, 0.264
Interviewer 39	0.115	-0.080, 0.309
Interviewer 40	0.091	-0.085, 0.266
Interviewer 41	0.058	-0.109, 0.225
Interviewer 42	0.250	0.047, 0.453
Interviewer 43	0.138	-0.022, 0.297
Interviewer 44	−0.006	-0.139, 0.127
Interviewer 45	−0.062	-0.211, 0.087
Interviewer 46	−0.008	-0.157, 0.141
Interviewer 47	0.004	-0.132, 0.140
Interviewer 48	0.089	-0.067, 0.244
Interviewer 49	0.095	-0.068, 0.257
Interviewer 50	−0.028	-0.196, 0.140
Interviewer 51	−0.027	-0.204, 0.151
Interviewer 52	0.042	-0.097, 0.182
Interviewer 53	0.019	-0.074, 0.113
Interviewer 54	−0.003	-0.140, 0.135
Interviewer 55	0.047	-0.093, 0.186
Interviewer 56	0.109	-0.046, 0.264
Interviewer 57	0.148	-0.001, 0.297
Interviewer 58	0.140	-0.033, 0.312
Interviewer 59	0.127	-0.038, 0.292
Interviewer 60	0.217	0.031, 0.403
Interviewer 61	0.039	-0.106, 0.185
Interviewer 62	0.069	-0.061, 0.199
Interviewer 63	0.026	-0.113, 0.166
Interviewer 64	0.002	-0.117, 0.121
Interviewer 65	−0.041	-0.154, 0.073
Interviewer 66	0.106	-0.040, 0.251
Interviewer 67	0.002	-0.141, 0.145
Interviewer 68	0.082	-0.065, 0.229
Interviewer 69	−0.067	-0.205, 0.071
Interviewer 70	−0.049	-0.165, 0.067
Interviewer 71	−0.077	-0.205, 0.051
Interviewer 72	0.010	-0.112, 0.132
Interviewer 73	0.022	-0.103, 0.147
Interviewer 74	0.052	-0.069, 0.173
Interviewer 75	0.079	-0.105, 0.263
First day	0.030	0.008, 0.053

N=7170, Censored cases=1618

ρ =-0.027, χ^2 =0.007, p=0.935

Table 12. Zimbabwe 2005 Consent Regression Marginals, Male

	dy/dx	95% CI
Age category		
20 – 24	0.017	-0.018, 0.052
25 – 29	0.131	0.079, 0.184
30 – 34	0.226	0.158, 0.294
35 – 39	0.239	0.167, 0.310
40 – 44	0.238	0.169, 0.306
45 – 49	0.199	0.132, 0.266
50 – 54	0.163	0.097, 0.228
Wealth quintile		
2nd	0.003	-0.026, 0.033
3rd	0.003	-0.029, 0.035
4th	0.030	-0.011, 0.071
5th	0.002	-0.048, 0.052
Mean education	−0.002	-0.005, 0.002
Location		
Small city	−0.052	-0.129, 0.024
Town	−0.017	-0.065, 0.031
Countryside	−0.026	-0.071, 0.019
Region		
Mashonaland central	−0.003	-0.052, 0.046
Mashonaland east	0.004	-0.041, 0.048
Mashonaland west	0.008	-0.035, 0.051
Matebeleland north	−0.021	-0.100, 0.058
Matebeleland south	0.002	-0.072, 0.075
Midlands	−0.013	-0.058, 0.033
Masvingo	0.005	-0.033, 0.042
Religion		
Roman Catholic	0.011	-0.024, 0.047
Protestant	−0.015	-0.049, 0.019
Pentecostal	−0.016	-0.053, 0.021
Apostolic sect	−0.015	-0.047, 0.017
None	0.023	-0.006, 0.051
Other	−0.024	-0.073, 0.025
Knows someone who died of AIDS	−0.019	-0.038, -0.001
Would care for relative with AIDS	0.005	-0.015, 0.026
Age at first sex		
≤15 yrs	0.068	0.023, 0.113
>15 yrs	0.060	0.019, 0.101
Married	−0.003	-0.038, 0.033
Ever tested for HIV	0.015	-0.007, 0.038
Number of partners in last 12 months		
One	−0.022	-0.065, 0.020

continued on next page

continued from previous page

	dy/dx	95% CI
Multiple	−0.044	-0.106, 0.018
STD in last 12 months	0.089	0.056, 0.122
Smokes tobacco	0.020	-0.001, 0.041
High risk sex in last 12 months	0.011	-0.028, 0.049
Condom use at last sex	0.019	-0.009, 0.047
Interview language		
Ndebele	0.031	-0.035, 0.097
English	0.037	-0.039, 0.113
Age category		
20 – 24	−0.041	-0.075, -0.006
25 – 29	−0.064	-0.105, -0.022
30 – 34	−0.054	-0.100, -0.008
35 – 39	−0.063	-0.114, -0.013
40 – 44	−0.042	-0.095, 0.012
45 – 49	−0.051	-0.109, 0.007
50 – 54	−0.061	-0.125, 0.002
Wealth quintile		
2nd	0.008	-0.031, 0.048
3rd	−0.021	-0.060, 0.018
4th	−0.038	-0.080, 0.004
5th	−0.076	-0.131, -0.022
Mean education	−0.003	-0.008, 0.002
Location		
Small city	−0.185	-0.354, -0.015
Town	−0.109	-0.205, -0.013
Countryside	0.037	-0.058, 0.132
Region		
Mashonaland central	−0.146	-0.236, -0.056
Mashonaland east	0.014	-0.096, 0.124
Mashonaland west	0.036	-0.068, 0.140
Matebeleland north	−0.173	-0.357, 0.012
Matebeleland south	0.003	-0.193, 0.198
Midlands	0.107	-0.036, 0.250
Masvingo	0.031	-0.053, 0.114
Religion		
Roman Catholic	−0.018	-0.069, 0.033
Protestant	0.000	-0.047, 0.048
Pentecostal	−0.023	-0.070, 0.024
Apostolic sect	−0.051	-0.096, -0.005
None	−0.035	-0.080, 0.009
Other	−0.007	-0.070, 0.056
Knows someone who died of AIDS	0.006	-0.016, 0.028
Would care for relative with AIDS	0.012	-0.012, 0.037
Age at first sex		
≤15 yrs	0.008	-0.040, 0.055

continued on next page

continued from previous page

	dy/dx	95% CI
>15 yrs	0.001	-0.040, 0.041
Married	0.008	-0.041, 0.058
Ever tested for HIV	−0.002	-0.030, 0.025
Number of partners in last 12 months		
One	0.007	-0.051, 0.065
Multiple	0.026	-0.060, 0.112
STD in last 12 months	0.040	-0.005, 0.084
Smokes tobacco	0.013	-0.012, 0.038
High risk sex in last 12 months	0.011	-0.040, 0.062
Condom use at last sex	0.028	-0.009, 0.064
Interview language		
Ndebele	−0.054	-0.157, 0.048
English	−0.130	-0.218, -0.043
Interviewer 2	−0.080	-0.232, 0.071
Interviewer 3	−0.030	-0.140, 0.080
Interviewer 4	0.041	-0.108, 0.191
Interviewer 5	0.115	0.002, 0.229
Interviewer 6	0.212	0.072, 0.352
Interviewer 7	−0.018	-0.137, 0.101
Interviewer 8	0.221	0.046, 0.397
Interviewer 9	0.277	0.100, 0.453
Interviewer 10	0.243	0.073, 0.414
Interviewer 11	0.165	-0.009, 0.340
Interviewer 12	0.203	0.017, 0.388
Interviewer 13	0.130	-0.052, 0.312
Interviewer 14	0.051	-0.136, 0.239
Interviewer 15	0.091	-0.074, 0.255
Interviewer 16	0.065	-0.125, 0.254
Interviewer 17	0.137	-0.033, 0.308
Interviewer 18	−0.063	-0.188, 0.061
Interviewer 19	−0.001	-0.138, 0.135
Interviewer 20	0.087	-0.039, 0.214
Interviewer 21	−0.132	-0.321, 0.058
Interviewer 22	0.092	-0.108, 0.293
Interviewer 23	−0.098	-0.279, 0.082
Interviewer 24	0.153	-0.035, 0.341
Interviewer 25	0.159	-0.032, 0.351
Interviewer 26	−0.042	-0.175, 0.091
Interviewer 27	−0.095	-0.215, 0.025
Interviewer 28	0.035	-0.107, 0.177
Interviewer 29	−0.005	-0.115, 0.105
Interviewer 30	0.013	-0.120, 0.146
Interviewer 31	0.037	-0.093, 0.167
Interviewer 32	0.095	-0.045, 0.235
Interviewer 33	0.158	0.009, 0.307

continued on next page

continued from previous page

	dy/dx	95% CI
Interviewer 34	0.063	-0.080, 0.206
Interviewer 35	−0.006	-0.135, 0.122
Interviewer 36	−0.040	-0.161, 0.080
Interviewer 37	−0.093	-0.212, 0.026
Interviewer 38	−0.103	-0.224, 0.018
Interviewer 39	−0.001	-0.123, 0.121
Interviewer 40	−0.043	-0.197, 0.111
Interviewer 41	−0.029	-0.134, 0.076

N=6929, Censored cases=1537

ρ =-0.043, χ^2 =0.037, p=0.848

Table 13. Lesotho 2009 – 2010 Contact Regression Marginals, Female

	dy/dx	95% CI
Age category		
20 – 24	0.266	0.214, 0.317
25 – 29	0.395	0.342, 0.448
30 – 34	0.443	0.391, 0.494
35 – 39	0.440	0.383, 0.497
40 – 44	0.382	0.322, 0.441
45 – 49	0.345	0.285, 0.405
Wealth quintile		
2nd	0.067	0.024, 0.111
3rd	0.093	0.040, 0.146
4th	0.114	0.060, 0.169
5th	0.050	-0.016, 0.116
Mean education	−0.010	-0.016, -0.004
Rural	−0.035	-0.081, 0.011
Region		
Leribe	0.068	0.001, 0.134
Berea	0.058	-0.025, 0.140
Maseru	0.110	0.047, 0.173
Mafeteng	0.018	-0.064, 0.099
Mohale's Hoek	0.098	0.031, 0.165
Quthing	0.095	0.017, 0.172
Qacha's-Nek	0.096	0.026, 0.165
Mokhotlong	0.062	-0.015, 0.140
Thaba-Tseka	0.046	-0.024, 0.117
Age category		
20 – 24	0.002	-0.004, 0.009
25 – 29	−0.006	-0.013, 0.001
30 – 34	−0.006	-0.013, 0.001
35 – 39	−0.003	-0.011, 0.005
40 – 44	0.004	-0.005, 0.014
45 – 49	0.002	-0.005, 0.010
Wealth quintile		
2nd	0.001	-0.006, 0.009
3rd	−0.000	-0.009, 0.008
4th	−0.006	-0.014, 0.002
5th	−0.012	-0.022, -0.003
Mean education	0.001	-0.000, 0.002
Rural	0.008	0.001, 0.014
Region		
Leribe	0.018	0.000, 0.037
Berea	−0.019	-0.045, 0.007
Maseru	−0.016	-0.046, 0.013

continued on next page

continued from previous page

	dy/dx	95% CI
Mafeteng	0.016	-0.013, 0.046
Mohale's Hoek	−0.008	-0.036, 0.020
Quthing	0.001	-0.032, 0.034
Qacha's-Nek	−0.007	-0.042, 0.028
Mokhotlong	0.004	-0.029, 0.037
Thaba-Tseka	−0.010	-0.043, 0.023
Interviewer 2	0.063	-0.002, 0.128
Interviewer 3	0.049	-0.014, 0.111
Interviewer 4	0.042	-0.021, 0.105
Interviewer 5	0.043	-0.019, 0.104
Interviewer 6	0.044	-0.019, 0.106
Interviewer 7	0.073	0.007, 0.139
Interviewer 8	0.058	-0.010, 0.125
Interviewer 9	0.054	-0.007, 0.115
Interviewer 10	0.059	-0.004, 0.121
Interviewer 11	0.057	-0.005, 0.120
Interviewer 12	0.057	-0.004, 0.117
Interviewer 13	0.071	0.010, 0.132
Interviewer 14	0.080	0.019, 0.142
Interviewer 15	0.248	0.174, 0.322
Interviewer 16	0.067	0.003, 0.131
Interviewer 17	0.071	0.004, 0.139
Interviewer 18	0.074	0.006, 0.142
Interviewer 19	0.233	0.158, 0.309
Interviewer 20	0.041	-0.023, 0.105
Interviewer 21	0.060	-0.005, 0.126
Interviewer 22	0.239	0.162, 0.316
Interviewer 23	0.239	0.163, 0.316
Interviewer 24	0.059	-0.007, 0.125
Interviewer 25	0.234	0.095, 0.373
Interviewer 26	0.074	0.005, 0.143
Interviewer 27	0.243	0.166, 0.319
Interviewer 28	0.057	-0.007, 0.121
Interviewer 29	0.070	0.006, 0.135
Interviewer 30	0.070	0.006, 0.133
Interviewer 31	0.070	0.007, 0.133
Interviewer 32	0.233	0.156, 0.309
Interviewer 33	0.053	-0.013, 0.119
Interviewer 34	0.066	-0.002, 0.133
Interviewer 35	0.054	-0.012, 0.120
Interviewer 36	0.065	-0.002, 0.131
Interviewer 37	0.080	0.016, 0.144
Interviewer 38	0.070	0.008, 0.133
Interviewer 39	0.054	-0.009, 0.117
Interviewer 40	0.056	-0.004, 0.115

continued on next page

continued from previous page

	dy/dx	95% CI
Interviewer 41	0.081	0.016, 0.146
Interviewer 42	0.028	-0.033, 0.088
Interviewer 43	0.041	-0.019, 0.102
Interviewer 44	0.020	-0.040, 0.079
Interviewer 45	0.049	-0.012, 0.111
Interviewer 46	0.049	-0.011, 0.109
Interviewer 47	0.046	-0.012, 0.105
Interviewer 48	0.046	-0.013, 0.106
Interviewer 49	0.048	-0.010, 0.107
Interviewer 50	0.048	-0.009, 0.106
Interviewer 51	0.064	0.001, 0.128
Interviewer 52	0.065	0.000, 0.130
Interviewer 53	0.060	-0.004, 0.124
Interviewer 54	0.065	-0.003, 0.133
Interviewer 55	0.052	-0.012, 0.116
Interviewer 56	0.063	-0.003, 0.128
Interviewer 57	0.057	-0.007, 0.121
Interviewer 58	0.064	0.000, 0.128
Interviewer 59	0.084	0.018, 0.149
Interviewer 60	0.071	0.006, 0.137
Interviewer 61	0.082	0.015, 0.150
Interviewer 62	0.064	-0.001, 0.128
Interviewer 63	0.082	0.012, 0.151
Interviewer 64	0.067	-0.001, 0.135
Interviewer 65	0.074	0.004, 0.145
Interviewer 66	0.043	-0.025, 0.111
Interviewer 67	0.084	0.023, 0.144
Interviewer 68	0.086	0.030, 0.143
Interviewer 69	0.063	0.005, 0.121
Interviewer 70	0.073	0.017, 0.130
Interviewer 71	0.065	0.008, 0.122
Interviewer 72	0.091	0.029, 0.153
Interviewer 73	0.085	0.022, 0.147
Interviewer 74	0.062	0.001, 0.123
Interviewer 75	0.251	0.176, 0.325
Interviewer 76	0.076	0.015, 0.137
First day	0.002	-0.003, 0.007

N=4076, Censored cases=252

ρ =-0.403, χ^2 =1.403, p=0.236

Table 14. Lesotho 2009 – 2010 Consent Regression Marginals, Female

	dy/dx	95% CI
Age category		
20 – 24	0.248	0.180, 0.316
25 – 29	0.404	0.339, 0.469
30 – 34	0.450	0.383, 0.517
35 – 39	0.445	0.372, 0.518
40 – 44	0.380	0.307, 0.454
45 – 49	0.336	0.262, 0.410
Wealth quintile		
2nd	0.061	0.013, 0.108
3rd	0.079	0.024, 0.134
4th	0.097	0.041, 0.154
5th	0.039	-0.029, 0.107
Mean education	−0.011	-0.017, -0.004
Rural	−0.026	-0.070, 0.018
Region		
Leribe	0.063	-0.013, 0.139
Berea	0.029	-0.054, 0.112
Maseru	0.070	-0.002, 0.141
Mafeteng	−0.012	-0.103, 0.079
Mohale's Hoek	0.069	-0.006, 0.144
Quthing	0.032	-0.054, 0.117
Qacha's-Nek	0.083	0.009, 0.157
Mokhotlong	0.038	-0.047, 0.122
Thaba-Tseka	0.013	-0.064, 0.090
Religion		
Lesotho Evangelical Church	−0.014	-0.056, 0.029
Pentecostal	−0.009	-0.047, 0.029
Knows someone who died of AIDS	0.044	0.006, 0.083
Would care for relative with AIDS	0.031	-0.040, 0.102
Age at first sex		
≤15 yrs	0.164	0.080, 0.248
>15 yrs	0.106	0.021, 0.191
Married	−0.140	-0.198, -0.081
Ever tested for HIV	−0.012	-0.047, 0.024
Number of partners in last 12 months		
One	0.094	0.023, 0.165
Multiple	0.077	-0.029, 0.183
STD in last 12 months	0.085	0.043, 0.127
Smokes tobacco	0.188	-0.131, 0.508
High risk sex in last 12 months	0.034	-0.027, 0.096
Condom use at last sex	0.095	0.059, 0.132
Age category		

continued on next page

continued from previous page

	dy/dx	95% CI
20 – 24	−0.000	-0.000, -0.000
25 – 29	−0.000	-0.000, -0.000
30 – 34	−0.000	-0.000, -0.000
35 – 39	−0.000	-0.000, -0.000
40 – 44	−0.000	-0.000, -0.000
45 – 49	−0.000	-0.000, -0.000
Wealth quintile		
2nd	−0.000	-0.000, -0.000
3rd	−0.000	-0.000, -0.000
4th	−0.000	-0.000, -0.000
5th	−0.001	-0.001, -0.001
Mean education	−0.000	-0.000, -0.000
Rural	0.000	0.000, 0.000
Region		
Leribe	0.001	0.001, 0.001
Berea	−0.001	-0.001, -0.001
Maseru	−0.001	-0.001, -0.001
Mafeteng	0.001	0.001, 0.001
Mohale's Hoek	−0.000	-0.000, -0.000
Quthing	0.001	0.001, 0.001
Qacha's-Nek	−0.000	-0.000, -0.000
Mokhotlong	0.000	0.000, 0.000
Thaba-Tseka	−0.000	-0.000, -0.000
Religion		
Lesotho Evangelical Church	−0.000	-0.000, -0.000
Pentecostal	−0.000	-0.000, -0.000
Knows someone who died of AIDS	0.000	0.000, 0.000
Would care for relative with AIDS	0.001	0.001, 0.001
Age at first sex		
≤15 yrs	0.000	0.000, 0.000
>15 yrs	0.000	0.000, 0.000
Married	−0.000	-0.000, -0.000
Ever tested for HIV	0.000	0.000, 0.000
Number of partners in last 12 months		
One	0.000	0.000, 0.000
Multiple	0.001	0.001, 0.001
STD in last 12 months	0.000	0.000, 0.000
Smokes tobacco	0.021	0.021, 0.021
High risk sex in last 12 months	−0.000	-0.000, -0.000
Condom use at last sex	0.000	0.000, 0.000
Interviewer 2	−0.007	-0.007, -0.007
Interviewer 3	−0.007	-0.007, -0.007
Interviewer 4	−0.007	-0.007, -0.007
Interviewer 5	−0.008	-0.008, -0.008
Interviewer 6	−0.007	-0.007, -0.007

continued on next page

continued from previous page

	dy/dx	95% CI
Interviewer 7	−0.006	-0.006, -0.006
Interviewer 8	−0.007	-0.007, -0.007
Interviewer 9	−0.007	-0.007, -0.007
Interviewer 10	−0.007	-0.007, -0.007
Interviewer 11	−0.006	-0.006, -0.006
Interviewer 12	−0.007	-0.007, -0.007
Interviewer 13	−0.006	-0.006, -0.006
Interviewer 14	−0.006	-0.006, -0.006
Interviewer 15	0.003	0.003, 0.003
Interviewer 16	−0.006	-0.006, -0.006
Interviewer 17	−0.007	-0.007, -0.007
Interviewer 18	0.001	0.001, 0.001
Interviewer 19	0.013	0.013, 0.013
Interviewer 20	−0.008	-0.008, -0.008
Interviewer 21	−0.007	-0.007, -0.007
Interviewer 22	0.002	0.002, 0.002
Interviewer 23	0.002	0.002, 0.002
Interviewer 24	−0.007	-0.007, -0.007
Interviewer 25	0.024	0.024, 0.024
Interviewer 26	0.002	0.002, 0.002
Interviewer 27	0.005	0.005, 0.005
Interviewer 28	−0.006	-0.006, -0.006
Interviewer 29	−0.006	-0.006, -0.006
Interviewer 30	−0.006	-0.006, -0.006
Interviewer 31	−0.006	-0.006, -0.006
Interviewer 32	0.001	0.001, 0.001
Interviewer 33	−0.007	-0.007, -0.007
Interviewer 34	−0.007	-0.007, -0.007
Interviewer 35	−0.007	-0.007, -0.007
Interviewer 36	−0.007	-0.007, -0.007
Interviewer 37	0.001	0.001, 0.001
Interviewer 38	−0.007	-0.007, -0.007
Interviewer 39	−0.007	-0.007, -0.007
Interviewer 40	−0.007	-0.007, -0.007
Interviewer 41	−0.007	-0.007, -0.007
Interviewer 42	−0.008	-0.008, -0.008
Interviewer 43	−0.007	-0.007, -0.007
Interviewer 44	−0.008	-0.008, -0.008
Interviewer 45	−0.008	-0.008, -0.008
Interviewer 46	−0.007	-0.007, -0.007
Interviewer 47	−0.008	-0.008, -0.008
Interviewer 48	−0.007	-0.007, -0.007
Interviewer 49	−0.007	-0.007, -0.007
Interviewer 50	−0.007	-0.007, -0.007
Interviewer 51	−0.006	-0.006, -0.006

continued on next page

continued from previous page

	dy/dx	95% CI
Interviewer 52	−0.006	-0.006, -0.006
Interviewer 53	−0.007	-0.007, -0.007
Interviewer 54	−0.006	-0.006, -0.006
Interviewer 55	−0.007	-0.007, -0.007
Interviewer 56	−0.006	-0.006, -0.006
Interviewer 57	−0.007	-0.007, -0.007
Interviewer 58	−0.006	-0.006, -0.006
Interviewer 59	−0.006	-0.006, -0.006
Interviewer 60	−0.007	-0.007, -0.007
Interviewer 61	−0.006	-0.006, -0.006
Interviewer 62	−0.006	-0.006, -0.006
Interviewer 63	−0.006	-0.006, -0.006
Interviewer 64	−0.007	-0.007, -0.007
Interviewer 65	−0.006	-0.006, -0.006
Interviewer 66	−0.007	-0.007, -0.007
Interviewer 67	−0.005	-0.005, -0.005
Interviewer 68	−0.006	-0.006, -0.006
Interviewer 69	−0.006	-0.006, -0.006
Interviewer 70	0.006	0.006, 0.006
Interviewer 71	−0.007	-0.007, -0.007
Interviewer 72	0.004	0.004, 0.004
Interviewer 73	−0.006	-0.006, -0.006
Interviewer 74	−0.006	-0.006, -0.006
Interviewer 75	0.002	0.002, 0.002
Interviewer 76	−0.006	-0.006, -0.006

N=3669, Censored cases=154

ρ =-0.843, χ^2 =1.442, p=0.230

Table 15. Lesotho 2009 – 2010 Contact Regression Marginals, Male

	dy/dx	95% CI
Age category		
20 – 24	0.078	0.019, 0.136
25 – 29	0.232	0.161, 0.304
30 – 34	0.351	0.262, 0.440
35 – 39	0.317	0.226, 0.407
40 – 44	0.369	0.272, 0.466
45 – 49	0.301	0.207, 0.396
50 – 54	0.256	0.168, 0.345
55 – 59	0.254	0.178, 0.331
Wealth quintile		
2nd	0.038	-0.002, 0.078
3rd	0.068	0.022, 0.114
4th	0.077	0.022, 0.131
5th	0.083	0.016, 0.150
Mean education	−0.006	-0.010, -0.002
Rural	0.004	-0.036, 0.044
Region		
Leribe	0.094	0.032, 0.156
Berea	0.059	0.002, 0.116
Maseru	0.091	0.031, 0.150
Mafeteng	0.082	0.024, 0.141
Mohale's Hoek	0.074	0.004, 0.143
Quthing	0.081	0.022, 0.140
Qacha's-Nek	0.073	0.004, 0.143
Mokhotlong	0.059	-0.001, 0.118
Thaba-Tseka	0.068	0.005, 0.131
Age category		
20 – 24	−0.056	-0.090, -0.022
25 – 29	−0.056	-0.091, -0.020
30 – 34	−0.053	-0.092, -0.015
35 – 39	−0.068	-0.108, -0.028
40 – 44	−0.075	-0.119, -0.031
45 – 49	−0.086	-0.126, -0.045
50 – 54	−0.076	-0.121, -0.030
55 – 59	−0.040	-0.089, 0.010
Wealth quintile		
2nd	−0.003	-0.040, 0.034
3rd	−0.012	-0.052, 0.028
4th	−0.056	-0.098, -0.014
5th	−0.062	-0.112, -0.011
Mean education	0.003	0.000, 0.007
Rural	0.035	-0.001, 0.072

continued on next page

continued from previous page

	dy/dx	95% CI
Region		
Leribe	0.104	0.039, 0.168
Berea	−0.019	-0.117, 0.080
Maseru	0.005	-0.055, 0.065
Mafeteng	0.033	-0.031, 0.097
Mohale's Hoek	0.052	-0.015, 0.119
Quthing	0.088	0.019, 0.156
Qacha's-Nek	0.054	-0.013, 0.121
Mokhotlong	0.092	0.022, 0.161
Thaba-Tseka	0.072	0.006, 0.139
Interviewer 2	0.108	0.006, 0.209
Interviewer 3	0.014	-0.083, 0.110
Interviewer 4	0.002	-0.080, 0.085
Interviewer 5	0.087	-0.010, 0.184
Interviewer 6	0.089	-0.010, 0.189
Interviewer 7	−0.039	-0.105, 0.027
Interviewer 8	−0.018	-0.098, 0.061
Interviewer 9	0.078	-0.013, 0.168
Interviewer 10	0.208	0.067, 0.348
Interviewer 11	0.064	-0.040, 0.169
Interviewer 12	0.014	-0.073, 0.101
Interviewer 13	0.152	0.011, 0.294
Interviewer 14	0.046	-0.042, 0.135
Interviewer 15	−0.104	-0.166, -0.042
Interviewer 16	0.022	-0.071, 0.116
Interviewer 17	0.014	-0.085, 0.114
Interviewer 18	0.038	-0.060, 0.137
Interviewer 19	0.109	-0.022, 0.240
Interviewer 20	0.145	-0.012, 0.301
Interviewer 21	0.067	-0.046, 0.180
Interviewer 22	0.085	-0.009, 0.179
Interviewer 23	0.053	-0.051, 0.157
Interviewer 24	0.226	0.079, 0.373
Interviewer 25	0.048	-0.034, 0.129
Interviewer 26	0.156	0.049, 0.262
Interviewer 27	0.101	0.001, 0.200
First day	0.014	-0.009, 0.037

N=3477, Censored cases=411

ρ =0.006, χ^2 =0.000, p=0.988

Table 16. Lesotho 2009 – 2010 Consent Regression Marginals, Male

	dy/dx	95% CI
Age category		
20 – 24	0.073	0.012, 0.134
25 – 29	0.239	0.179, 0.300
30 – 34	0.356	0.282, 0.430
35 – 39	0.333	0.257, 0.408
40 – 44	0.371	0.290, 0.452
45 – 49	0.330	0.247, 0.414
50 – 54	0.253	0.168, 0.338
55 – 59	0.266	0.183, 0.348
Wealth quintile		
2nd	0.052	0.006, 0.097
3rd	0.063	0.015, 0.111
4th	0.077	0.021, 0.133
5th	0.116	0.046, 0.186
Mean education	−0.009	-0.013, -0.005
Rural	0.010	-0.032, 0.053
Region		
Leribe	0.084	0.014, 0.155
Berea	0.040	-0.026, 0.106
Maseru	0.057	-0.007, 0.121
Mafeteng	0.064	-0.005, 0.134
Mohale's Hoek	0.051	-0.022, 0.124
Quthing	0.047	-0.021, 0.116
Qacha's-Nek	0.045	-0.035, 0.126
Mokhotlong	0.028	-0.050, 0.106
Thaba-Tseka	0.057	-0.011, 0.126
Religion		
Lesotho Evangelical Church	0.015	-0.021, 0.051
Pentecostal	0.002	-0.035, 0.038
Would care for relative with AIDS	−0.004	-0.049, 0.042
Age at first sex		
≤15 yrs	−0.032	-0.118, 0.054
>15 yrs	−0.045	-0.127, 0.037
Married	0.025	-0.020, 0.069
Ever tested for HIV	0.042	0.011, 0.072
Number of partners in last 12 months		
One	0.021	-0.040, 0.082
Multiple	0.046	-0.030, 0.123
STD in last 12 months	0.086	0.050, 0.123
Smokes tobacco	0.013	-0.016, 0.042
High risk sex in last 12 months	0.029	-0.020, 0.078
Condom use at last sex	0.074	0.039, 0.108

continued on next page

continued from previous page

	dy/dx	95% CI
Age category		
20 – 24	−0.011	-0.023, 0.001
25 – 29	−0.009	-0.022, 0.005
30 – 34	−0.014	-0.029, 0.001
35 – 39	−0.012	-0.028, 0.004
40 – 44	−0.025	-0.042, -0.009
45 – 49	−0.019	-0.037, -0.002
50 – 54	−0.016	-0.035, 0.003
55 – 59	−0.016	-0.033, 0.000
Wealth quintile		
2nd	−0.005	-0.017, 0.008
3rd	−0.003	-0.017, 0.011
4th	−0.020	-0.034, -0.006
5th	−0.022	-0.038, -0.005
Mean education	0.000	-0.001, 0.001
Rural	0.012	0.002, 0.022
Region		
Leribe	0.036	0.014, 0.057
Berea	0.015	-0.010, 0.040
Maseru	0.019	-0.000, 0.039
Mafeteng	0.015	-0.007, 0.037
Mohale's Hoek	0.022	0.001, 0.043
Quthing	0.033	0.007, 0.059
Qacha's-Nek	0.012	-0.008, 0.033
Mokhotlong	0.061	0.033, 0.089
Thaba-Tseka	0.012	-0.010, 0.033
Religion		
Lesotho Evangelical Church	0.003	-0.006, 0.012
Pentecostal	−0.010	-0.019, -0.001
Would care for relative with AIDS	0.001	-0.010, 0.012
Age at first sex		
≤15 yrs	0.011	-0.005, 0.027
>15 yrs	0.011	-0.006, 0.027
Married	0.001	-0.012, 0.013
Ever tested for HIV	0.014	0.007, 0.020
Number of partners in last 12 months		
One	−0.009	-0.025, 0.006
Multiple	0.000	-0.020, 0.021
STD in last 12 months	0.007	-0.005, 0.018
Smokes tobacco	−0.002	-0.009, 0.006
High risk sex in last 12 months	0.006	-0.006, 0.019
Condom use at last sex	−0.002	-0.010, 0.006
Interviewer 2	0.713	0.566, 0.861
Interviewer 3	0.004	-0.026, 0.034
Interviewer 4	0.009	-0.019, 0.037

continued on next page

continued from previous page

	dy/dx	95% CI
Interviewer 5	0.029	-0.006, 0.064
Interviewer 6	0.013	-0.009, 0.034
Interviewer 7	−0.015	-0.060, 0.029
Interviewer 8	0.029	-0.005, 0.063
Interviewer 9	0.028	-0.005, 0.061
Interviewer 10	0.021	-0.017, 0.059
Interviewer 11	0.022	-0.016, 0.060
Interviewer 12	−0.035	-0.053, -0.018
Interviewer 13	−0.015	-0.040, 0.010
Interviewer 14	−0.007	-0.035, 0.022
Interviewer 15	0.257	0.197, 0.317
Interviewer 16	0.024	-0.012, 0.061
Interviewer 17	0.015	-0.024, 0.055
Interviewer 18	−0.003	-0.030, 0.024
Interviewer 19	0.004	-0.021, 0.030
Interviewer 20	−0.021	-0.050, 0.009
Interviewer 21	0.025	-0.009, 0.060
Interviewer 22	0.005	-0.021, 0.031
Interviewer 23	0.010	-0.019, 0.039

N=2792, Censored cases=192

ρ =-0.463, χ^2 =2.047, p=0.152

Table 17. Lesotho 2004 Contact Regression Marginals, Female

	dy/dx	95% CI
Age category		
20 – 24	0.213	0.159, 0.267
25 – 29	0.360	0.293, 0.426
30 – 34	0.361	0.299, 0.424
35 – 39	0.372	0.307, 0.438
40 – 44	0.263	0.193, 0.333
45 – 49	0.127	0.053, 0.201
Wealth quintile		
2nd	0.089	0.030, 0.148
3rd	0.077	0.019, 0.135
4th	0.091	0.025, 0.157
5th	0.059	-0.013, 0.132
Mean education	−0.007	-0.014, -0.000
Rural	−0.072	-0.114, -0.030
Region		
Leribe	0.047	-0.022, 0.116
Berea	0.017	-0.059, 0.092
Maseru	0.045	-0.037, 0.128
Mafeteng	0.033	-0.049, 0.116
Mohale's Hoek	−0.028	-0.102, 0.045
Quithing	0.045	-0.029, 0.119
Qasha's Nek	0.040	-0.047, 0.126
Mokhotlong	−0.033	-0.126, 0.060
Thaba-Tseka	−0.011	-0.095, 0.074
Age category		
20 – 24	0.022	-0.015, 0.058
25 – 29	−0.018	-0.056, 0.019
30 – 34	0.055	0.012, 0.098
35 – 39	0.018	-0.025, 0.060
40 – 44	−0.036	-0.077, 0.005
45 – 49	−0.015	-0.055, 0.026
Wealth quintile		
2nd	−0.023	-0.073, 0.027
3rd	−0.037	-0.094, 0.021
4th	−0.054	-0.110, 0.002
5th	−0.087	-0.146, -0.029
Mean education	−0.007	-0.012, -0.001
Rural	0.027	-0.016, 0.071
Region		
Leribe	−0.075	-0.186, 0.037
Berea	−0.107	-0.243, 0.030
Maseru	−0.067	-0.222, 0.088

continued on next page

continued from previous page

	dy/dx	95% CI
Mafeteng	−0.003	-0.179, 0.172
Mohale's Hoek	0.061	-0.139, 0.261
Quithing	−0.882	-1.115, -0.648
Qasha's Nek	0.047	-0.155, 0.248
Mokhotlong	−0.084	-0.277, 0.109
Thaba-Tseka	−0.261	-0.699, 0.177
Interviewer 2	0.140	-0.080, 0.360
Interviewer 3	−0.068	-0.294, 0.158
Interviewer 4	0.065	-0.154, 0.285
Interviewer 5	−0.084	-0.316, 0.147
Interviewer 6	0.071	-0.154, 0.295
Interviewer 7	−0.063	-0.279, 0.153
Interviewer 8	0.068	-0.156, 0.292
Interviewer 9	0.211	0.008, 0.414
Interviewer 10	0.102	-0.100, 0.304
Interviewer 11	0.222	-0.019, 0.463
Interviewer 12	0.227	0.019, 0.434
Interviewer 13	0.101	-0.075, 0.278
Interviewer 14	0.190	0.018, 0.362
Interviewer 15	0.009	-0.162, 0.181
Interviewer 16	0.077	-0.101, 0.256
Interviewer 17	−0.191	-0.366, -0.017
Interviewer 18	0.020	-0.122, 0.162
Interviewer 19	−0.084	-0.234, 0.067
Interviewer 20	−0.078	-0.243, 0.086
Interviewer 21	−0.028	-0.201, 0.144
Interviewer 22	0.013	-0.155, 0.182
Interviewer 23	−0.050	-0.208, 0.107
Interviewer 24	−0.118	-0.278, 0.042
Interviewer 25	0.130	-0.041, 0.302
Interviewer 26	0.070	-0.104, 0.243
Interviewer 27	0.116	-0.060, 0.291
Interviewer 28	0.076	-0.105, 0.256
Interviewer 29	−0.093	-0.287, 0.100
Interviewer 30	−0.095	-0.300, 0.110
Interviewer 31	−0.105	-0.296, 0.086
Interviewer 32	−0.116	-0.309, 0.077
Interviewer 33	0.949	0.714, 1.184
Interviewer 34	0.938	0.715, 1.162
Interviewer 35	0.978	0.759, 1.196
Interviewer 36	0.932	0.702, 1.163
Interviewer 37	−0.044	-0.248, 0.160
Interviewer 38	0.096	-0.152, 0.343
Interviewer 39	−0.041	-0.243, 0.162
Interviewer 40	0.107	-0.106, 0.319

continued on next page

continued from previous page

	dy/dx	95% CI
Interviewer 41	0.308	-0.154, 0.770
Interviewer 42	0.329	-0.124, 0.783
Interviewer 43	0.284	-0.172, 0.740
Interviewer 44	0.178	-0.278, 0.635
Interviewer 45	0.220	-0.032, 0.473
Interviewer 46	0.233	0.023, 0.444
Interviewer 47	0.097	-0.147, 0.341
Interviewer 48	0.006	-0.221, 0.234
Interviewer 49	0.048	-0.167, 0.262
First day	0.015	-0.013, 0.043

N=3688, Censored cases=662

ρ =-0.095, χ^2 =0.103, p=0.748

Table 18. Lesotho 2004 Consent Regression Marginals, Female

	dy/dx	95% CI
Age category		
20 – 24	0.166	0.107, 0.224
25 – 29	0.307	0.237, 0.378
30 – 34	0.310	0.239, 0.382
35 – 39	0.310	0.236, 0.384
40 – 44	0.204	0.128, 0.280
45 – 49	0.056	-0.026, 0.138
Wealth quintile		
2nd	0.080	0.019, 0.141
3rd	0.072	0.013, 0.132
4th	0.088	0.023, 0.154
5th	0.059	-0.012, 0.131
Mean education	−0.011	-0.018, -0.004
Rural	−0.050	-0.093, -0.006
Region		
Leribe	0.041	-0.028, 0.110
Berea	0.023	-0.053, 0.098
Maseru	0.032	-0.044, 0.107
Mafeteng	0.023	-0.058, 0.103
Mohale's Hoek	−0.031	-0.104, 0.042
Quithing	0.027	-0.045, 0.100
Qasha's Nek	0.001	-0.085, 0.088
Mokhotlong	−0.050	-0.135, 0.034
Thaba-Tseka	0.008	-0.076, 0.093
Knows someone who died of AIDS	−0.023	-0.064, 0.019
Would care for relative with AIDS	−0.016	-0.063, 0.031
Age at first sex		
≤15	0.163	0.074, 0.252
>15	0.179	0.096, 0.262
Married	−0.125	-0.188, -0.063
Ever tested for HIV	0.068	0.018, 0.118
Number of partners in last 12 months		
One	0.047	-0.029, 0.123
Multiple	0.114	-0.012, 0.240
STD in last 12 months	0.126	0.084, 0.169
Smokes tobacco	−0.123	-0.567, 0.320
High risk sex in last 12 months	0.031	-0.040, 0.101
Condom use at last sex	0.020	-0.033, 0.073
Age category		
20 – 24	−0.002	-0.002, -0.002
25 – 29	−0.017	-0.017, -0.017
30 – 34	0.004	0.004, 0.004

continued on next page

continued from previous page

	dy/dx	95% CI
35 – 39	−0.023	-0.023, -0.023
40 – 44	−0.057	-0.057, -0.057
45 – 49	−0.045	-0.045, -0.045
Wealth quintile		
2nd	−0.015	-0.015, -0.015
3rd	−0.031	-0.031, -0.031
4th	−0.048	-0.048, -0.048
5th	−0.071	-0.071, -0.071
edu0	−0.010	-0.010, -0.010
Rural	0.044	0.044, 0.044
Region		
Leribe	−0.095	-0.095, -0.095
Berea	−0.123	-0.123, -0.123
Maseru	−0.080	-0.080, -0.080
Mafeteng	−0.021	-0.021, -0.021
Mohale's Hoek	0.041	0.041, 0.041
Quithing	−0.708	-0.708, -0.708
Qasha's Nek	−0.029	-0.029, -0.029
Mokhotlong	−0.131	-0.131, -0.131
Thaba-Tseka	0.779	0.779, 0.779
Knows someone who died of AIDS	0.010	0.010, 0.010
Would care for relative with AIDS	0.010	0.010, 0.010
Age at first sex		
≤15	0.027	0.027, 0.027
>15	0.037	0.037, 0.037
Married	0.025	0.025, 0.025
Ever tested for HIV	−0.004	-0.004, -0.004
Number of partners in last 12 months		
One	−0.057	-0.057, -0.057
Multiple	−0.102	-0.102, -0.102
STD in last 12 months	0.033	0.033, 0.033
Smokes tobacco	−0.083	-0.083, -0.083
High risk sex in last 12 months	0.047	0.047, 0.047
Condom use at last sex	−0.017	-0.017, -0.017
Interviewer 2	0.087	0.087, 0.087
Interviewer 3	−0.069	-0.069, -0.069
Interviewer 4	0.031	0.031, 0.031
Interviewer 5	−0.066	-0.066, -0.066
Interviewer 6	−0.025	-0.025, -0.025
Interviewer 7	−0.041	-0.041, -0.041
Interviewer 8	0.051	0.051, 0.051
Interviewer 9	0.198	0.198, 0.198
Interviewer 10	0.148	0.148, 0.148
Interviewer 11	0.212	0.212, 0.212
Interviewer 12	0.198	0.198, 0.198

continued on next page

continued from previous page

	dy/dx	95% CI
Interviewer 13	0.062	0.062, 0.062
Interviewer 14	0.182	0.182, 0.182
Interviewer 15	0.024	0.024, 0.024
Interviewer 16	0.097	0.097, 0.097
Interviewer 17	−0.112	-0.112, -0.112
Interviewer 18	0.038	0.038, 0.038
Interviewer 19	−0.070	-0.070, -0.070
Interviewer 20	−0.034	-0.034, -0.034
Interviewer 21	−0.045	-0.045, -0.045
Interviewer 22	−0.009	-0.009, -0.009
Interviewer 23	0.018	0.018, 0.018
Interviewer 24	−0.078	-0.078, -0.078
Interviewer 25	0.118	0.118, 0.118
Interviewer 26	0.103	0.103, 0.103
Interviewer 27	0.170	0.170, 0.170
Interviewer 28	0.124	0.124, 0.124
Interviewer 29	−0.072	-0.072, -0.072
Interviewer 30	−0.083	-0.083, -0.083
Interviewer 31	−0.113	-0.113, -0.113
Interviewer 32	−0.082	-0.082, -0.082
Interviewer 33	0.759	0.759, 0.759
Interviewer 34	0.705	0.705, 0.705
Interviewer 35	0.778	0.778, 0.778
Interviewer 36	0.838	0.838, 0.838
Interviewer 37	0.039	0.039, 0.039
Interviewer 38	0.092	0.092, 0.092
Interviewer 39	−0.018	-0.018, -0.018
Interviewer 40	0.094	0.094, 0.094
Interviewer 41	−0.795	-0.795, -0.795
Interviewer 42	−0.712	-0.712, -0.712
Interviewer 43	−0.802	-0.802, -0.802
Interviewer 44	−0.905	-0.905, -0.905
Interviewer 45	0.235	0.235, 0.235
Interviewer 46	0.185	0.185, 0.185
Interviewer 47	0.097	0.097, 0.097
Interviewer 48	0.054	0.054, 0.054
Interviewer 49	0.149	0.149, 0.149

N=3198, Censored cases=452

ρ =0.033, χ^2 =0.017, p=0.898

Table 19. Lesotho 2004 Contact Regression Marginals, Male

	dy/dx	95% CI
Age category		
20 – 24	0.174	0.042, 0.307
25 – 29	0.267	0.080, 0.454
30 – 34	0.353	0.117, 0.588
35 – 39	0.344	0.108, 0.580
40 – 44	0.325	0.093, 0.556
45 – 49	0.239	0.065, 0.413
50 – 54	0.230	0.071, 0.389
55 – 59	0.202	0.055, 0.350
Wealth quintile		
2nd	0.001	-0.042, 0.044
3rd	0.040	-0.016, 0.096
4th	0.035	-0.025, 0.094
5th	−0.018	-0.077, 0.041
Mean education	−0.002	-0.006, 0.001
Rural	−0.044	-0.096, 0.009
Region		
Leribe	0.080	-0.004, 0.163
Berea	0.068	0.005, 0.130
Maseru	0.057	-0.070, 0.184
Mafeteng	0.064	-0.001, 0.129
Mohale's Hoek	0.060	-0.017, 0.138
Quithing	0.056	-0.017, 0.128
Qasha's Nek	0.046	-0.021, 0.113
Mokhotlong	−0.004	-0.074, 0.066
Thaba-Tseka	0.057	-0.009, 0.123
Age category		
20 – 24	−0.034	-0.085, 0.017
25 – 29	−0.061	-0.112, -0.009
30 – 34	−0.026	-0.082, 0.029
35 – 39	−0.063	-0.124, -0.002
40 – 44	−0.057	-0.128, 0.014
45 – 49	−0.074	-0.139, -0.009
50 – 54	−0.008	-0.087, 0.071
55 – 59	−0.026	-0.103, 0.051
Wealth quintile		
2nd	−0.014	-0.070, 0.042
3rd	−0.052	-0.106, 0.003
4th	−0.071	-0.132, -0.010
5th	−0.125	-0.196, -0.055
Mean education	−0.002	-0.007, 0.003
Rural	0.031	-0.025, 0.086

continued on next page

continued from previous page

	dy/dx	95% CI
Region		
Leribe	−0.060	-0.180, 0.061
Berea	0.027	-0.107, 0.160
Maseru	−0.021	-0.150, 0.108
Mafeteng	0.073	-0.076, 0.222
Mohale's Hoek	0.058	-0.118, 0.234
Quithing	−0.058	-0.245, 0.130
Qasha's Nek	0.178	0.017, 0.339
Mokhotlong	0.004	-0.407, 0.415
Thaba-Tseka	0.145	-0.052, 0.342
Interviewer 2	0.390	0.250, 0.530
Interviewer 3	0.036	-0.143, 0.215
Interviewer 4	0.231	0.090, 0.372
Interviewer 5	0.099	-0.032, 0.231
Interviewer 6	0.193	0.062, 0.324
Interviewer 7	0.253	0.082, 0.425
Interviewer 8	0.201	0.073, 0.329
Interviewer 9	0.257	0.109, 0.405
Interviewer 10	0.252	0.101, 0.403
Interviewer 11	0.252	0.098, 0.405
Interviewer 12	0.045	-0.083, 0.173
Interviewer 13	0.099	-0.057, 0.256
Interviewer 14	0.134	0.029, 0.239
Interviewer 15	−0.073	-0.173, 0.027
Interviewer 16	−0.063	-0.199, 0.074
Interviewer 17	0.131	0.005, 0.257
Interviewer 18	−0.104	-0.256, 0.048
Interviewer 19	0.358	0.180, 0.536
Interviewer 20	0.212	0.052, 0.371
Interviewer 21	0.219	0.056, 0.381
Interviewer 22	0.161	0.013, 0.310
Interviewer 23	0.107	-0.077, 0.291
Interviewer 24	−0.028	-0.202, 0.146
Interviewer 25	−0.031	-0.211, 0.149
Interviewer 26	−0.092	-0.258, 0.074
Interviewer 27	0.324	0.137, 0.511
Interviewer 28	0.183	0.006, 0.359
Interviewer 29	0.164	0.002, 0.326
Interviewer 30	0.161	-0.002, 0.324
Interviewer 31	0.130	-0.038, 0.298
Interviewer 32	0.124	-0.031, 0.279
Interviewer 33	0.086	-0.123, 0.296
Interviewer 34	0.005	-0.172, 0.182
Interviewer 35	0.067	-0.136, 0.270
Interviewer 36	0.030	-0.180, 0.241

continued on next page

continued from previous page

	dy/dx	95% CI
Interviewer 37	0.190	-0.247, 0.628
Interviewer 38	0.247	-0.243, 0.737
Interviewer 39	0.168	-0.270, 0.605
Interviewer 40	0.110	-0.302, 0.523
Interviewer 41	0.209	0.064, 0.354
First day	0.003	-0.031, 0.038

N=3178, Censored cases=933

ρ =0.278, χ^2 =0.187, p=0.665

Table 20. Lesotho 2004 Consent Regression Marginals, Male

	dy/dx	95% CI
Age category		
20 – 24	0.167	0.090, 0.245
25 – 29	0.254	0.164, 0.344
30 – 34	0.357	0.250, 0.463
35 – 39	0.340	0.236, 0.443
40 – 44	0.337	0.227, 0.447
45 – 49	0.214	0.112, 0.316
50 – 54	0.211	0.113, 0.310
55 – 59	0.208	0.101, 0.315
Wealth quintile		
2nd	−0.001	-0.054, 0.053
3rd	0.041	-0.015, 0.097
4th	0.033	-0.027, 0.092
5th	−0.017	-0.088, 0.053
Mean education	−0.003	-0.008, 0.001
Rural	−0.059	-0.111, -0.007
Region		
Leribe	0.089	0.017, 0.161
Berea	0.081	0.010, 0.151
Maseru	0.073	-0.022, 0.169
Mafeteng	0.064	-0.015, 0.144
Mohale's Hoek	0.057	-0.012, 0.126
Quithing	0.039	-0.033, 0.112
Qasha's Nek	0.029	-0.048, 0.105
Mokhotlong	−0.025	-0.110, 0.060
Thaba-Tseka	0.083	0.017, 0.150
Knows someone who died of AIDS	−0.034	-0.074, 0.006
Would care for relative with AIDS	0.001	-0.040, 0.042
Age at first sex		
≤15	0.051	-0.045, 0.147
>15	0.069	-0.024, 0.163
Married	0.034	-0.014, 0.081
Ever tested for HIV	0.062	0.013, 0.111
Number of partners last 12 months		
Single	−0.011	-0.075, 0.054
Multiple	−0.019	-0.102, 0.065
STD in last 12 months	0.085	0.037, 0.133
Smokes tobacco	0.019	-0.022, 0.059
High risk sex in last 12 months	0.037	-0.011, 0.085
Age category		
20 – 24	−0.012	-0.064, 0.040
25 – 29	−0.027	-0.085, 0.030

continued on next page

continued from previous page

	dy/dx	95% CI
30 – 34	−0.021	-0.090, 0.048
35 – 39	−0.024	-0.098, 0.050
40 – 44	−0.030	-0.099, 0.040
45 – 49	−0.069	-0.141, 0.004
50 – 54	0.003	-0.076, 0.082
55 – 59	−0.055	-0.136, 0.026
Wealth quintile		
2nd	−0.028	-0.084, 0.028
3rd	−0.061	-0.115, -0.008
4th	−0.051	-0.109, 0.006
5th	−0.106	-0.170, -0.042
Mean education	−0.007	-0.011, -0.002
Rural	0.071	0.029, 0.113
Region		
Leribe	0.004	-0.098, 0.106
Berea	0.030	-0.074, 0.133
Maseru	0.000	-0.106, 0.106
Mafeteng	0.067	-0.047, 0.181
Mohale's Hoek	0.044	-0.080, 0.167
Quithing	−0.914	-1.113, -0.715
Qasha's Nek	0.094	-0.050, 0.237
Mokhotlong	0.119	0.007, 0.231
Thaba-Tseka	0.104	-0.011, 0.219
Knows someone who died of AIDS	−0.005	-0.036, 0.027
Would care for relative with AIDS	−0.003	-0.043, 0.037
Age at first sex		
≤15	−0.020	-0.085, 0.044
>15	−0.039	-0.105, 0.026
Married	−0.024	-0.073, 0.024
Ever tested for HIV	−0.022	-0.064, 0.020
Number of partners last 12 months		
Single	0.061	0.000, 0.121
Multiple	0.073	-0.003, 0.149
STD in last 12 months	0.086	0.029, 0.144
Smokes tobacco	0.012	-0.027, 0.051
High risk sex in last 12 months	−0.018	-0.066, 0.031
Interviewer 2	0.213	0.087, 0.339
Interviewer 3	0.033	-0.111, 0.177
Interviewer 4	0.204	0.083, 0.324
Interviewer 5	−0.061	-0.160, 0.038
Interviewer 6	0.191	0.069, 0.312
Interviewer 7	0.215	0.094, 0.336
Interviewer 8	0.122	0.010, 0.234
Interviewer 9	0.196	0.093, 0.299
Interviewer 10	−0.003	-0.125, 0.119

continued on next page

continued from previous page

	dy/dx	95% CI
Interviewer 11	0.011	-0.079, 0.101
Interviewer 12	0.092	-0.053, 0.237
Interviewer 13	−0.168	-0.268, -0.068
Interviewer 14	−0.067	-0.172, 0.039
Interviewer 15	−0.087	-0.189, 0.016
Interviewer 16	−0.058	-0.180, 0.065
Interviewer 17	0.225	0.088, 0.363
Interviewer 18	0.247	0.098, 0.396
Interviewer 19	0.121	0.007, 0.236
Interviewer 20	0.063	-0.081, 0.206
Interviewer 21	−0.044	-0.158, 0.071
Interviewer 22	−0.144	-0.252, -0.036
Interviewer 23	1.114	0.922, 1.306
Interviewer 24	1.131	0.926, 1.337
Interviewer 25	1.026	0.862, 1.189
Interviewer 26	1.038	0.869, 1.207
Interviewer 27	0.145	-0.011, 0.301
Interviewer 28	0.046	-0.105, 0.198
Interviewer 29	0.026	-0.129, 0.181
Interviewer 30	0.141	0.005, 0.276
Interviewer 31	0.050	-0.082, 0.183
Interviewer 32	−0.043	-0.155, 0.070
Interviewer 33	−0.110	-0.251, 0.032
Interviewer 34	0.049	-0.044, 0.142

N=2484, Censored cases=470

ρ =-0.077, χ^2 =0.055, p=0.814
