# Supplementary material for: Validation, Replication, and Sensitivity Testing of Heckman-Type Selection Models to Adjust Estimates of HIV Prevalence
Source: PLoS One. 2014 Nov 17;9(11):e112563. doi: 10.1371/journal.pone.0112563 (PMC4234405; doi:10.1371/journal.pone.0112563)
Supplement: Tables S2 — Regressions for the Agincourt health and demographic surveillance system. (PDF) [file pone.0112563.s002.pdf]

# Agincourt Regression Estimation Results Tables

**Table 1.** Model M1 Estimation Results

| Variable | Coefficient | (Std. Err.) |
|---|---|---|
| Outcome Equation: $[F{:}I]$ | | |
| age = 20 | -0.174 | (0.230) |
| age = 25 | -0.339 | (0.225) |
| age = 30 | -0.303 | (0.216) |
| age = 35 | -0.514* | (0.210) |
| age = 40 | -0.551* | (0.222) |
| age = 45 | -0.580** | (0.219) |
| age = 50 | 0.016 | (0.299) |
| age = 55 | -0.371 | (0.251) |
| age = 60 | -0.276 | (0.263) |
| age = 65 | -0.341 | (0.261) |
| age = 70 | -0.543* | (0.274) |
| age = 75 | -0.556* | (0.282) |
| age = 80 | -0.289 | (0.317) |
| sex = 1 | -0.039 | (0.245) |
| age = 20 and sex = 1 | -0.041 | (0.313) |
| age = 25 and sex = 1 | -0.433 | (0.313) |
| age = 30 and sex = 1 | -0.595† | (0.319) |
| age = 35 and sex = 1 | -0.317 | (0.301) |
| age = 40 and sex = 1 | -0.369 | (0.328) |
| age = 45 and sex = 1 | -0.158 | (0.317) |
| age = 50 and sex = 1 | -0.966* | (0.377) |
| age = 55 and sex = 1 | -0.285 | (0.371) |
| age = 60 and sex = 1 | -0.222 | (0.354) |
| age = 65 and sex = 1 | -0.355 | (0.352) |
| age = 70 and sex = 1 | 0.242 | (0.394) |
| age = 75 and sex = 1 | 5.229** | (0.331) |
| age = 80 and sex = 1 | -0.363 | (0.429) |
| village = 2 | -0.021 | (0.179) |
| village = 3 | 0.003 | (0.156) |
| village = 4 | -0.017 | (0.185) |
| village = 5 | 0.363† | (0.191) |
| village = 6 | -0.140 | (0.182) |
| village = 7 | -0.173 | (0.216) |
| village = 8 | 0.109 | (0.169) |
| village = 9 | -0.249† | (0.147) |
| village = 10 | 0.444** | (0.161) |
| village = 11 | 0.066 | (0.145) |
| village = 12 | 0.052 | (0.188) |
| village = 13 | -0.073 | (0.169) |
| village = 14 | -0.450† | (0.252) |
| village = 15 | 0.213 | (0.231) |
| village = 16 | -0.020 | (0.143) |
| village = 17 | -0.150 | (0.257) |
| village = 18 | 0.228 | (0.287) |
| village = 19 | 0.476 | (0.310) |

*Continued on next page...*

... *table 1 continued*

| Variable | Coefficient | (Std. Err.) |
|---|---|---|
| village = 20 | -0.043 | (0.254) |
| village = 21 | 0.081 | (0.305) |
| migration = 1 | -0.147$^{\dagger}$ | (0.078) |
| SES quintile = 2 | 0.052 | (0.129) |
| SES quintile = 3 | -0.176 | (0.124) |
| SES quintile = 4 | -0.268$^{*}$ | (0.119) |
| SES quintile = 5 | -0.290$^{*}$ | (0.119) |
| Intercept | 2.327$^{**}$ | (0.241) |
| Selection Equation: $[F]$ | | |
| age = 20 | -0.322$^{**}$ | (0.108) |
| age = 25 | -0.351$^{**}$ | (0.106) |
| age = 30 | -0.203$^{\dagger}$ | (0.108) |
| age = 35 | 0.000 | (0.110) |
| age = 40 | -0.146 | (0.116) |
| age = 45 | 0.005 | (0.120) |
| age = 50 | 0.145 | (0.149) |
| age = 55 | 0.107 | (0.147) |
| age = 60 | 0.511$^{**}$ | (0.171) |
| age = 65 | 0.443$^{**}$ | (0.167) |
| age = 70 | 0.376$^{*}$ | (0.189) |
| age = 75 | 0.578$^{**}$ | (0.221) |
| age = 80 | 0.358$^{\dagger}$ | (0.201) |
| sex = 1 | 0.164 | (0.126) |
| age = 20 and sex = 1 | -0.681$^{**}$ | (0.154) |
| age = 25 and sex = 1 | -0.835$^{**}$ | (0.153) |
| age = 30 and sex = 1 | -0.999$^{**}$ | (0.155) |
| age = 35 and sex = 1 | -0.971$^{**}$ | (0.156) |
| age = 40 and sex = 1 | -0.973$^{**}$ | (0.166) |
| age = 45 and sex = 1 | -0.994$^{**}$ | (0.170) |
| age = 50 and sex = 1 | -0.940$^{**}$ | (0.203) |
| age = 55 and sex = 1 | -0.984$^{**}$ | (0.203) |
| age = 60 and sex = 1 | -0.895$^{**}$ | (0.223) |
| age = 65 and sex = 1 | -0.805$^{**}$ | (0.227) |
| age = 70 and sex = 1 | -0.678$^{**}$ | (0.248) |
| age = 75 and sex = 1 | -0.713$^{*}$ | (0.320) |
| age = 80 and sex = 1 | 0.081 | (0.329) |
| village = 2 | -0.499$^{**}$ | (0.112) |
| village = 3 | -0.047 | (0.098) |
| village = 4 | -0.028 | (0.116) |
| village = 5 | -0.166 | (0.114) |
| village = 6 | 0.111 | (0.115) |
| village = 7 | -0.168 | (0.133) |
| village = 8 | -0.138 | (0.099) |
| village = 9 | -0.301$^{**}$ | (0.101) |
| village = 10 | -0.129 | (0.101) |
| village = 11 | -0.189$^{*}$ | (0.088) |
| village = 12 | -0.020 | (0.137) |
| village = 13 | -0.188 | (0.121) |
| village = 14 | -0.381$^{*}$ | (0.152) |

*Continued on next page...*

*... table 1 continued*

| Variable | Coefficient | (Std. Err.) |
|---|---|---|
| village = 15 | 0.049 | (0.110) |
| village = 16 | 0.117 | (0.115) |
| village = 17 | -0.189 | (0.141) |
| village = 18 | -0.286$^{\dagger}$ | (0.169) |
| village = 19 | 0.223 | (0.180) |
| village = 20 | -0.049 | (0.177) |
| village = 21 | -0.040 | (0.154) |
| migration = 1 | -0.173$^{**}$ | (0.045) |
| SES quintile = 2 | 0.043 | (0.071) |
| SES quintile = 3 | 0.039 | (0.070) |
| SES quintile = 4 | 0.044 | (0.070) |
| SES quintile = 5 | -0.058 | (0.070) |
| Intercept | 1.139$^{**}$ | (0.122) |
| $\rho$ | -0.215 | (0.288) |

Significance levels : † : 10% ∗ : 5% ∗∗ : 1%

**Table 2.** Model M2 Estimation Results

| **Variable** | **Coefficient** | **(Std. Err.)** |
|---|---|---|
| Outcome Equation: $[F{:}I{:}T]$ | | |
| age = 20 | -0.503* | (0.230) |
| age = 25 | -0.803** | (0.221) |
| age = 30 | -0.605** | (0.226) |
| age = 35 | -0.662** | (0.225) |
| age = 40 | -0.694** | (0.236) |
| age = 45 | -0.406 | (0.256) |
| age = 50 | -0.482† | (0.264) |
| age = 55 | -0.393 | (0.269) |
| age = 60 | -0.581* | (0.254) |
| age = 65 | -0.754** | (0.246) |
| age = 70 | -0.485 | (0.303) |
| age = 75 | -0.472 | (0.327) |
| age = 80 | -0.416 | (0.330) |
| sex = 1 | -0.323 | (0.244) |
| age = 20 and sex = 1 | 0.095 | (0.304) |
| age = 25 and sex = 1 | -0.144 | (0.296) |
| age = 30 and sex = 1 | -0.341 | (0.304) |
| age = 35 and sex = 1 | -0.154 | (0.296) |
| age = 40 and sex = 1 | -0.130 | (0.318) |
| age = 45 and sex = 1 | -0.262 | (0.323) |
| age = 50 and sex = 1 | -0.289 | (0.363) |
| age = 55 and sex = 1 | 0.119 | (0.394) |
| age = 60 and sex = 1 | 0.199 | (0.342) |
| age = 65 and sex = 1 | 0.899* | (0.420) |
| age = 70 and sex = 1 | 0.544 | (0.420) |
| age = 75 and sex = 1 | 0.169 | (0.470) |
| age = 80 and sex = 1 | 0.281 | (0.484) |
| village = 2 | -0.202 | (0.217) |
| village = 3 | -0.048 | (0.168) |
| village = 4 | -0.377† | (0.196) |
| village = 5 | -0.056 | (0.183) |
| village = 6 | -0.253 | (0.184) |
| village = 7 | 0.362 | (0.225) |
| village = 8 | -0.018 | (0.155) |
| village = 9 | -0.375* | (0.178) |
| village = 10 | 0.224 | (0.175) |
| village = 11 | 0.100 | (0.153) |
| village = 12 | 0.593** | (0.226) |
| village = 13 | -0.089 | (0.179) |
| village = 14 | 0.476 | (0.293) |
| village = 15 | 0.165 | (0.221) |
| village = 16 | -0.249 | (0.175) |
| village = 17 | 0.033 | (0.245) |
| village = 18 | 0.048 | (0.267) |
| village = 19 | -0.008 | (0.285) |
| village = 20 | 0.151 | (0.327) |
| village = 21 | 0.106 | (0.213) |

*Continued on next page...*

*... table 2 continued*

| Variable | Coefficient | (Std. Err.) |
|---|---|---|
| migration = 1 | -0.017 | (0.083) |
| SES quintile = 2 | -0.036 | (0.119) |
| SES quintile = 3 | -0.156 | (0.119) |
| SES quintile = 4 | -0.414** | (0.118) |
| SES quintile = 5 | -0.494** | (0.117) |
| Intercept | 2.430** | (0.274) |
| Selection Equation: $[F{:}I]$ | | |
| age = 20 | -0.201 | (0.232) |
| age = 25 | -0.380† | (0.225) |
| age = 30 | -0.321 | (0.218) |
| age = 35 | -0.527* | (0.213) |
| age = 40 | -0.569* | (0.224) |
| age = 45 | -0.589** | (0.222) |
| age = 50 | 0.026 | (0.302) |
| age = 55 | -0.367 | (0.253) |
| age = 60 | -0.257 | (0.261) |
| age = 65 | -0.326 | (0.262) |
| age = 70 | -0.526† | (0.275) |
| age = 75 | -0.532† | (0.284) |
| age = 80 | -0.268 | (0.320) |
| sex = 1 | -0.029 | (0.249) |
| age = 20 and sex = 1 | -0.118 | (0.318) |
| age = 25 and sex = 1 | -0.524† | (0.300) |
| age = 30 and sex = 1 | -0.707* | (0.293) |
| age = 35 and sex = 1 | -0.407 | (0.287) |
| age = 40 and sex = 1 | -0.478 | (0.307) |
| age = 45 and sex = 1 | -0.253 | (0.304) |
| age = 50 and sex = 1 | -1.061** | (0.375) |
| age = 55 and sex = 1 | -0.381 | (0.360) |
| age = 60 and sex = 1 | -0.264 | (0.353) |
| age = 65 and sex = 1 | -0.403 | (0.352) |
| age = 70 and sex = 1 | 0.205 | (0.398) |
| age = 75 and sex = 1 | 11.429 | (0.000) |
| age = 80 and sex = 1 | -0.368 | (0.433) |
| village = 2 | -0.079 | (0.177) |
| village = 3 | -0.003 | (0.157) |
| village = 4 | -0.038 | (0.188) |
| village = 5 | 0.357† | (0.193) |
| village = 6 | -0.136 | (0.182) |
| village = 7 | -0.188 | (0.215) |
| village = 8 | 0.103 | (0.171) |
| village = 9 | -0.280* | (0.142) |
| village = 10 | 0.442** | (0.162) |
| village = 11 | 0.053 | (0.144) |
| village = 12 | 0.053 | (0.190) |
| village = 13 | -0.088 | (0.170) |
| village = 14 | -0.493* | (0.233) |
| village = 15 | 0.224 | (0.235) |
| village = 16 | -0.020 | (0.144) |

*Continued on next page...*

*... table 2 continued*

| **Variable** | **Coefficient** | **(Std. Err.)** |
|---|---|---|
| village = 17 | -0.169 | (0.262) |
| village = 18 | 0.211 | (0.290) |
| village = 19 | 0.489 | (0.308) |
| village = 20 | -0.045 | (0.258) |
| village = 21 | 0.088 | (0.311) |
| migration = 1 | -0.162* | (0.075) |
| SES quintile = 2 | 0.058 | (0.131) |
| SES quintile = 3 | -0.174 | (0.125) |
| SES quintile = 4 | -0.272* | (0.120) |
| SES quintile = 5 | -0.296* | (0.119) |
| Intercept | 2.301** | (0.241) |
| $\rho$ | 0.414 | (0.230) |

Significance levels : † : 10% $*$ : 5% $**$ : 1%

**Table 3.** Model M3 Estimation Results

| Variable | Coefficient | (Std. Err.) |
|---|---|---|
| Outcome Equation: $[H]$ | | |
| age = 20 | 1.025** | (0.153) |
| age = 25 | 1.391** | (0.152) |
| age = 30 | 1.422** | (0.150) |
| age = 35 | 1.521** | (0.149) |
| age = 40 | 1.257** | (0.158) |
| age = 45 | 1.222** | (0.159) |
| age = 50 | 1.039** | (0.176) |
| age = 55 | 1.018** | (0.175) |
| age = 60 | 0.567** | (0.188) |
| age = 65 | 0.447* | (0.207) |
| age = 70 | 0.388† | (0.219) |
| age = 75 | 0.064 | (0.254) |
| age = 80 | -0.614 | (0.405) |
| sex = 1 | -0.991** | (0.334) |
| age = 20 and sex = 1 | 0.078 | (0.362) |
| age = 25 and sex = 1 | 0.595† | (0.361) |
| age = 30 and sex = 1 | 1.082** | (0.351) |
| age = 35 and sex = 1 | 1.073** | (0.349) |
| age = 40 and sex = 1 | 1.206** | (0.361) |
| age = 45 and sex = 1 | 0.932* | (0.366) |
| age = 50 and sex = 1 | 1.164** | (0.383) |
| age = 55 and sex = 1 | 1.237** | (0.377) |
| age = 60 and sex = 1 | 1.267** | (0.383) |
| age = 65 and sex = 1 | 1.187** | (0.414) |
| age = 70 and sex = 1 | 0.686 | (0.433) |
| age = 75 and sex = 1 | 0.973† | (0.515) |
| age = 80 and sex = 1 | 1.140† | (0.643) |
| village = 2 | 0.178 | (0.183) |
| village = 3 | 0.114 | (0.121) |
| village = 4 | -0.012 | (0.152) |
| village = 5 | -0.114 | (0.135) |
| village = 6 | 0.056 | (0.144) |
| village = 7 | -0.095 | (0.155) |
| village = 8 | -0.082 | (0.125) |
| village = 9 | -0.057 | (0.131) |
| village = 10 | -0.217† | (0.121) |
| village = 11 | 0.047 | (0.113) |
| village = 12 | 0.073 | (0.157) |
| village = 13 | 0.001 | (0.141) |
| village = 14 | -0.025 | (0.179) |
| village = 15 | 0.034 | (0.141) |
| village = 16 | -0.329* | (0.146) |
| village = 17 | 0.129 | (0.154) |
| village = 18 | 0.226 | (0.197) |
| village = 19 | 0.195 | (0.212) |
| village = 20 | -0.268 | (0.217) |
| village = 21 | 0.664** | (0.193) |

*Continued on next page...*

*... table 3 continued*

| Variable | Coefficient | (Std. Err.) |
|---|---|---|
| migration = 1 | -0.024 | (0.058) |
| SES quintile = 2 | -0.160* | (0.081) |
| SES quintile = 3 | -0.070 | (0.085) |
| SES quintile = 4 | -0.070 | (0.098) |
| SES quintile = 5 | -0.351** | (0.110) |
| Intercept | -1.433** | (0.180) |
| Selection Equation: $[F{:}I{:}T]$ | | |
| age = 20 | -0.555* | (0.241) |
| age = 25 | -0.832** | (0.225) |
| age = 30 | -0.663** | (0.231) |
| age = 35 | -0.721** | (0.232) |
| age = 40 | -0.753** | (0.246) |
| age = 45 | -0.422 | (0.257) |
| age = 50 | -0.569* | (0.270) |
| age = 55 | -0.456† | (0.276) |
| age = 60 | -0.658* | (0.261) |
| age = 65 | -0.817** | (0.254) |
| age = 70 | -0.567† | (0.313) |
| age = 75 | -0.536 | (0.341) |
| age = 80 | -0.466 | (0.335) |
| sex = 1 | -0.377 | (0.251) |
| age = 20 and sex = 1 | 0.127 | (0.313) |
| age = 25 and sex = 1 | -0.031 | (0.299) |
| age = 30 and sex = 1 | -0.236 | (0.309) |
| age = 35 and sex = 1 | -0.027 | (0.301) |
| age = 40 and sex = 1 | 0.002 | (0.327) |
| age = 45 and sex = 1 | -0.185 | (0.332) |
| age = 50 and sex = 1 | -0.122 | (0.357) |
| age = 55 and sex = 1 | 0.223 | (0.414) |
| age = 60 and sex = 1 | 0.264 | (0.353) |
| age = 65 and sex = 1 | 0.976* | (0.442) |
| age = 70 and sex = 1 | 0.689 | (0.434) |
| age = 75 and sex = 1 | 0.175 | (0.482) |
| age = 80 and sex = 1 | 0.332 | (0.502) |
| village = 2 | -0.081 | (0.228) |
| village = 3 | -0.037 | (0.173) |
| village = 4 | -0.403* | (0.199) |
| village = 5 | -0.076 | (0.185) |
| village = 6 | -0.239 | (0.191) |
| village = 7 | 0.413† | (0.232) |
| village = 8 | -0.028 | (0.159) |
| village = 9 | -0.358* | (0.182) |
| village = 10 | 0.219 | (0.179) |
| village = 11 | 0.081 | (0.161) |
| village = 12 | 0.612** | (0.236) |
| village = 13 | -0.097 | (0.183) |
| village = 14 | 0.502† | (0.294) |
| village = 15 | 0.153 | (0.225) |
| village = 16 | -0.234 | (0.178) |

*Continued on next page...*

*... table 3 continued*

| **Variable** | **Coefficient** | **(Std. Err.)** |
|---|---|---|
| village = 17 | 0.012 | (0.250) |
| village = 18 | 0.091 | (0.286) |
| village = 19 | -0.039 | (0.285) |
| village = 20 | 0.179 | (0.338) |
| village = 21 | 0.123 | (0.222) |
| migration = 1 | 0.015 | (0.079) |
| SES quintile = 2 | -0.008 | (0.122) |
| SES quintile = 3 | -0.069 | (0.121) |
| SES quintile = 4 | -0.348$^{**}$ | (0.124) |
| SES quintile = 5 | -0.425$^{**}$ | (0.118) |
| fieldworker = 3713 | -0.123 | (0.168) |
| fieldworker = 3858 | -0.239 | (0.167) |
| fieldworker = 4680 | 0.289 | (0.227) |
| fieldworker = 5681 | 0.118 | (0.159) |
| fieldworker = 6547 | 0.463$^{*}$ | (0.180) |
| fieldworker = 6761 | 0.019 | (0.164) |
| fieldworker = 6963 | -0.286$^{\dagger}$ | (0.156) |
| fieldworker = 7683 | -0.287 | (0.191) |
| fieldworker = 8875 | -0.295$^{\dagger}$ | (0.166) |
| fieldworker = 9821 | 0.160 | (0.165) |
| Intercept | 2.547$^{**}$ | (0.299) |
| $\rho$ | -0.499 | (0.359) |

Significance levels : $\dagger$ : 10% $\quad *$ : 5% $\quad **$ : 1%

**Table 4.** Consent Model Estimation Results

| Variable | Coefficient | (Std. Err.) |
|---|---|---|
| Outcome Equation: $[H]$ | | |
| age = 20 | 1.024** | (0.154) |
| age = 25 | 1.388** | (0.155) |
| age = 30 | 1.423** | (0.151) |
| age = 35 | 1.534** | (0.150) |
| age = 40 | 1.269** | (0.162) |
| age = 45 | 1.249** | (0.160) |
| age = 50 | 1.031** | (0.177) |
| age = 55 | 1.028** | (0.175) |
| age = 60 | 0.554** | (0.190) |
| age = 65 | 0.429* | (0.213) |
| age = 70 | 0.396† | (0.226) |
| age = 75 | 0.079 | (0.264) |
| age = 80 | -0.622 | (0.414) |
| sex = 1 | -1.027** | (0.351) |
| age = 20 and sex = 1 | 0.098 | (0.376) |
| age = 25 and sex = 1 | 0.631† | (0.380) |
| age = 30 and sex = 1 | 1.139** | (0.371) |
| age = 35 and sex = 1 | 1.116** | (0.363) |
| age = 40 and sex = 1 | 1.270** | (0.376) |
| age = 45 and sex = 1 | 0.958* | (0.380) |
| age = 50 and sex = 1 | 1.237** | (0.404) |
| age = 55 and sex = 1 | 1.292** | (0.389) |
| age = 60 and sex = 1 | 1.319** | (0.395) |
| age = 65 and sex = 1 | 1.288** | (0.417) |
| age = 70 and sex = 1 | 0.720 | (0.449) |
| age = 75 and sex = 1 | 0.949† | (0.534) |
| age = 80 and sex = 1 | 1.202† | (0.654) |
| village = 2 | 0.172 | (0.185) |
| village = 3 | 0.114 | (0.122) |
| village = 4 | -0.035 | (0.154) |
| village = 5 | -0.136 | (0.136) |
| village = 6 | 0.049 | (0.146) |
| village = 7 | -0.065 | (0.152) |
| village = 8 | -0.091 | (0.126) |
| village = 9 | -0.058 | (0.142) |
| village = 10 | -0.231† | (0.129) |
| village = 11 | 0.050 | (0.115) |
| village = 12 | 0.088 | (0.158) |
| village = 13 | -0.002 | (0.142) |
| village = 14 | 0.040 | (0.176) |
| village = 15 | 0.029 | (0.146) |
| village = 16 | -0.350* | (0.147) |
| village = 17 | 0.141 | (0.155) |
| village = 18 | 0.218 | (0.200) |
| village = 19 | 0.180 | (0.216) |
| village = 20 | -0.262 | (0.217) |
| village = 21 | 0.668** | (0.199) |

*Continued on next page...*

*... table 4 continued*

| Variable | Coefficient | (Std. Err.) |
|---|---|---|
| migration = 1 | -0.014 | (0.059) |
| SES quintile = 2 | -0.164* | (0.082) |
| SES quintile = 3 | -0.066 | (0.088) |
| SES quintile = 4 | -0.074 | (0.112) |
| SES quintile = 5 | -0.359** | (0.131) |
| Intercept | -1.430** | (0.183) |
| Selection Equation: [$CT$:$CS$] | | |
| age = 20 | -0.416* | (0.185) |
| age = 25 | -0.676** | (0.178) |
| age = 30 | -0.522** | (0.177) |
| age = 35 | -0.672** | (0.178) |
| age = 40 | -0.702** | (0.185) |
| age = 45 | -0.566** | (0.188) |
| age = 50 | -0.334 | (0.220) |
| age = 55 | -0.428* | (0.209) |
| age = 60 | -0.505* | (0.207) |
| age = 65 | -0.669** | (0.204) |
| age = 70 | -0.585* | (0.231) |
| age = 75 | -0.574* | (0.244) |
| age = 80 | -0.403 | (0.261) |
| sex = 1 | -0.241 | (0.197) |
| age = 20 and sex = 1 | 0.027 | (0.250) |
| age = 25 and sex = 1 | -0.297 | (0.240) |
| age = 30 and sex = 1 | -0.540* | (0.239) |
| age = 35 and sex = 1 | -0.235 | (0.234) |
| age = 40 and sex = 1 | -0.316 | (0.252) |
| age = 45 and sex = 1 | -0.210 | (0.252) |
| age = 50 and sex = 1 | -0.609* | (0.287) |
| age = 55 and sex = 1 | -0.089 | (0.304) |
| age = 60 and sex = 1 | 0.008 | (0.278) |
| age = 65 and sex = 1 | 0.301 | (0.292) |
| age = 70 and sex = 1 | 0.504 | (0.327) |
| age = 75 and sex = 1 | 0.420 | (0.405) |
| age = 80 and sex = 1 | -0.010 | (0.363) |
| village = 2 | -0.097 | (0.168) |
| village = 3 | -0.014 | (0.134) |
| village = 4 | -0.274† | (0.160) |
| village = 5 | 0.116 | (0.150) |
| village = 6 | -0.226 | (0.152) |
| village = 7 | 0.065 | (0.187) |
| village = 8 | 0.019 | (0.134) |
| village = 9 | -0.361** | (0.133) |
| village = 10 | 0.357** | (0.138) |
| village = 11 | 0.074 | (0.122) |
| village = 12 | 0.276 | (0.174) |
| village = 13 | -0.078 | (0.143) |
| village = 14 | -0.188 | (0.215) |
| village = 15 | 0.210 | (0.182) |
| village = 16 | -0.161 | (0.135) |

*Continued on next page...*

*... table 4 continued*

| **Variable** | **Coefficient** | **(Std. Err.)** |
|---|---|---|
| village = 17 | -0.080 | (0.215) |
| village = 18 | 0.161 | (0.237) |
| village = 19 | 0.193 | (0.238) |
| village = 20 | 0.070 | (0.235) |
| village = 21 | 0.139 | (0.231) |
| migration = 1 | -0.076 | (0.063) |
| SES quintile = 2 | 0.027 | (0.102) |
| SES quintile = 3 | -0.147 | (0.102) |
| SES quintile = 4 | -0.359$^{**}$ | (0.100) |
| SES quintile = 5 | -0.435$^{**}$ | (0.097) |
| fieldworker = 3713 | -0.201 | (0.147) |
| fieldworker = 3858 | -0.266$^{\dagger}$ | (0.146) |
| fieldworker = 4680 | 0.008 | (0.184) |
| fieldworker = 5681 | 0.044 | (0.136) |
| fieldworker = 6547 | -0.085 | (0.158) |
| fieldworker = 6761 | -0.385$^{**}$ | (0.142) |
| fieldworker = 6963 | -0.207 | (0.136) |
| fieldworker = 7683 | -0.306$^{\dagger}$ | (0.161) |
| fieldworker = 8875 | -0.273$^{\dagger}$ | (0.141) |
| fieldworker = 9821 | -0.108 | (0.142) |
| Intercept | 2.295$^{**}$ | (0.231) |
| $\rho$ | -0.342 | (0.436) |

Significance levels : $\dagger$ : 10% $*$ : 5% $**$ : 1%

**Table 5.** Contact Model Estimation Results

| **Variable** | **Coefficient** | **(Std. Err.)** |
|---|---|---|
| Outcome Equation: $[H]$ | | |
| age = 20 | 0.886** | (0.137) |
| age = 25 | 1.198** | (0.137) |
| age = 30 | 1.246** | (0.135) |
| age = 35 | 1.386** | (0.131) |
| age = 40 | 1.157** | (0.140) |
| age = 45 | 1.114** | (0.138) |
| age = 50 | 0.901** | (0.156) |
| age = 55 | 0.914** | (0.153) |
| age = 60 | 0.583** | (0.158) |
| age = 65 | 0.570** | (0.160) |
| age = 70 | 0.511** | (0.181) |
| age = 75 | 0.324 | (0.198) |
| age = 80 | -0.098 | (0.235) |
| sex = 1 | -0.179 | (0.166) |
| age = 20 and sex = 1 | -0.430* | (0.210) |
| age = 25 and sex = 1 | 0.031 | (0.211) |
| age = 30 and sex = 1 | 0.368† | (0.215) |
| age = 35 and sex = 1 | 0.265 | (0.210) |
| age = 40 and sex = 1 | 0.432† | (0.228) |
| age = 45 and sex = 1 | 0.196 | (0.220) |
| age = 50 and sex = 1 | 0.532* | (0.246) |
| age = 55 and sex = 1 | 0.368 | (0.246) |
| age = 60 and sex = 1 | 0.406† | (0.235) |
| age = 65 and sex = 1 | 0.285 | (0.243) |
| age = 70 and sex = 1 | -0.129 | (0.275) |
| age = 75 and sex = 1 | 0.021 | (0.342) |
| age = 80 and sex = 1 | 0.483 | (0.334) |
| village = 2 | 0.167 | (0.148) |
| village = 3 | 0.124 | (0.107) |
| village = 4 | 0.125 | (0.133) |
| village = 5 | -0.109 | (0.116) |
| village = 6 | 0.148 | (0.128) |
| village = 7 | -0.047 | (0.144) |
| village = 8 | -0.070 | (0.108) |
| village = 9 | 0.145 | (0.112) |
| village = 10 | -0.281** | (0.105) |
| village = 11 | 0.007 | (0.098) |
| village = 12 | -0.013 | (0.131) |
| village = 13 | 0.046 | (0.119) |
| village = 14 | 0.118 | (0.174) |
| village = 15 | 0.003 | (0.131) |
| village = 16 | -0.115 | (0.119) |
| village = 17 | 0.146 | (0.143) |
| village = 18 | 0.097 | (0.171) |
| village = 19 | 0.104 | (0.181) |
| village = 20 | -0.184 | (0.183) |
| village = 21 | 0.521** | (0.176) |

*Continued on next page...*

*... table 5 continued*

| **Variable** | **Coefficient** | **(Std. Err.)** |
|---|---|---|
| Intercept | -1.428** | (0.142) |
| Selection Equation: $[CT]$ | | |
| age = 20 | -0.301** | (0.108) |
| age = 25 | -0.353** | (0.107) |
| age = 30 | -0.257* | (0.108) |
| age = 35 | -0.031 | (0.110) |
| age = 40 | -0.161 | (0.116) |
| age = 45 | 0.031 | (0.121) |
| age = 50 | 0.229 | (0.152) |
| age = 55 | 0.174 | (0.149) |
| age = 60 | 0.595** | (0.170) |
| age = 65 | 0.498** | (0.173) |
| age = 70 | 0.425* | (0.198) |
| age = 75 | 0.642** | (0.223) |
| age = 80 | 0.394* | (0.201) |
| sex = 1 | 0.183 | (0.127) |
| age = 20 and sex = 1 | -0.672** | (0.155) |
| age = 25 and sex = 1 | -0.810** | (0.154) |
| age = 30 and sex = 1 | -0.934** | (0.155) |
| age = 35 and sex = 1 | -0.932** | (0.156) |
| age = 40 and sex = 1 | -0.967** | (0.166) |
| age = 45 and sex = 1 | -0.973** | (0.169) |
| age = 50 and sex = 1 | -1.008** | (0.206) |
| age = 55 and sex = 1 | -0.963** | (0.204) |
| age = 60 and sex = 1 | -0.937** | (0.222) |
| age = 65 and sex = 1 | -0.796** | (0.231) |
| age = 70 and sex = 1 | -0.702** | (0.257) |
| age = 75 and sex = 1 | -0.817* | (0.320) |
| age = 80 and sex = 1 | 0.078 | (0.334) |
| fieldworker = 3713 | -1.049** | (0.162) |
| fieldworker = 3858 | -0.746** | (0.172) |
| fieldworker = 4680 | -1.541** | (0.169) |
| fieldworker = 5681 | -1.192** | (0.164) |
| fieldworker = 6547 | -1.301** | (0.163) |
| fieldworker = 6761 | -1.156** | (0.162) |
| fieldworker = 6963 | -1.141** | (0.163) |
| fieldworker = 7683 | -1.295** | (0.161) |
| fieldworker = 8875 | -1.118** | (0.161) |
| fieldworker = 9821 | -0.948** | (0.162) |
| Intercept | 2.019** | (0.169) |
| $\rho$ | 0.219 | (0.158) |

Significance levels : $\dagger$ : 10% $*$ : 5% $**$ : 1%
